# Real-World Experience with Venetoclax Therapeutic Drug Monitoring in Acute Myeloid Leukemia: Role of Posaconazole, Correlation with Safety and Efficacy

**DOI:** 10.32604/or.2026.078245

**Published:** 2026-07-16

**Authors:** Beatrice Sani, Alessandro Cignetti, Marta Leporati, Sara Sommariva, Marco Armenio, Valerio Tenace, Arianna Savi, Johanna Umurungi, Giovanni Fornari, Simone Busso, Alessandra Canevaro, Igor Bisognin, Silvia Marini, Michele Piana, Daniela Cilloni, Valentina Gaidano

**Affiliations:** 1 Department of Clinical and Biological Sciences, University of Turin, Turin, Italy; 2 University Division of Hematology and Cell Therapies, A.O. Ordine Mauriziano di Torino, Turin, Italy; 3 Laboratory of Analytical Chemistry and Kidney Stones, Analysis Laboratory, A.O. Ordine Mauriziano di Torino, Turin, Italy; 4 Department of Mathematics, University of Genoa, Genoa, Italy; 5 PrimisAI, Los Gatos, CA, USA

**Keywords:** Acute myeloid leukemia, venetoclax, therapeutic drug monitoring, toxicity

## Abstract

**Objectives:** Venetoclax (VEN) is approved for acute myeloid leukemia (AML) in association with azacitidine, in a 28-day schedule at a fixed dosage, which requires reduction if azoles are co-administered. The present study aims to evaluate VEN therapeutic drug monitoring (TDM) in a real-word setting, where the VEN schedule is frequently reduced, investigating: (*i*) the posaconazole impact, and (*ii*) whether VEN exposure correlates with safety and efficacy. **Methods:** We analyzed data from 43 AML patients treated with different VEN-containing regimens, for whom a near-trough VEN plasma concentration (Cmin) was determined at different timepoints (days 5-8-11-15-22-29) across different cycles (163 cycles, 290 determinations). The posaconazole impact was explored in the whole study population, while safety and efficacy were investigated only in patients treated with azacitidine-VEN, respectively in the safety (35 patients) and in the efficacy subset (29 patients at their first cycle). VEN exposure was expressed through multiple parameters, taking into account both VEN concentrations and the days of VEN administration. **Results:** Posaconazole was used in 40.5% of cycles and, despite dose adjustment, was associated with: (*i*) greater interpatient variability, (*ii*) higher VEN concentrations, (*iii*) delayed elimination, (*iv*) accumulation along the cycle, and (*v*) the need for VEN-dosage change. In the safety subset, VEN exposure correlated with neutropenia and its duration, Granulocyte Colony-Stimulating Factor requirement, platelet transfusions, cycle duration, and infections. Finally, no correlation was found between VEN exposure and response in the efficacy subset. **Conclusion:** VEN TDM appears valuable in clinical practice to reduce toxicity, especially in patients receiving posaconazole, where VEN exposure remains highly unpredictable.

## 1 Introduction

Venetoclax (VEN) is an oral, potent Bcl-2 inhibitor that has recently entered the therapeutic armamentarium against acute myeloid leukemia (AML). In association with hypomethylating agents, it has become the standard of care in unfit patients with *de novo* AML; moreover, it is frequently used in relapsed/refractory (R/R) patients both in association with hypomethylating agents and chemotherapy, and in a variety of exploratory settings (e.g., minimal residual disease-MRD eradication, bridge to transplant, maintenance, etc.).

The VIALE-A trial [1] demonstrated that in unfit patients with previously untreated AML, overall survival (OS) was longer with azacitidine + VEN (aza-ven) rather than azacitidine alone (14.7 vs. 9.6 months), but it also showed that the combination therapy was more frequently associated with neutropenia and infections. The infective aspect is so remarkable, especially in the unfit population, that it sometimes offsets the advantages of aza-ven in terms of response, resulting in OS rates comparable to azacitidine alone in some real-life studies [2]. In order to reduce toxicity, the clinical practice has been changing in the last years, leading to a reduction from 28 to 21, 14 or even 7 days of therapy; recent literature supports this approach, demonstrating a more favorable safety profile, similar antileukemic activity and, finally, similar or improved OS [3,4,5,6].

These reductions, however, are totally arbitrary, and do not account for the remarkable interpatient variability in VEN plasma concentrations that has been widely demonstrated [7,8,9,10]. Moreover, VEN plasma concentrations are influenced by multiple factors, such as food and drugs. Being extensively metabolized by CYP3A4, VEN concentrations are significantly increased in the presence of strong and moderate CYP3A4 inhibitors, such as posaconazole, voriconazole or isavuconazole. Agarwal et al. [11] showed that the exposure to VEN when administered with posaconazole was higher compared with the classical 400 mg/die dose given without posaconazole, even if VEN was reduced to 50 or 100 mg/die; moreover, it confirmed a prominent interpatient variability also in this setting, with a day 20 exposure (expressed as maximum concentration-Cmax) ranging from <0.5 to >6.5 μg/mL. This heterogeneous data led to different EMA (European Medical Agency) and FDA (Food and Drug Administration) Prescribing Information: the European label recommends to reduce the administered VEN dosage of at least 75 and 50% in the presence of strong and moderate CYP3A4 inhibitors, respectively; the American label recommends to reduce the VEN dosage to 70 mg when posaconazole is co-administered, 100 mg in the presence of other strong CYP3A4 inhibitors, and at least 50% for moderate inhibitors. The risk of overdose when CYP3A4 inhibitors, and in particular posaconazole, are co-administered can represent a vicious circle: posaconazole, in fact, is frequently used as prophylaxis in the first treatment cycles, when neutropenia is more frequent [12]: this could increase VEN plasma concentrations, prolonging the duration of cytopenias [13]; on the other hand, a low concentration of VEN could affect its antileukemic activity [8,14].

The interpatient variability, the need to frequently utilize antifungal agents, and the evidence that, *in vitro*, the inhibition of granulocyte forming units is dependent on VEN concentration [15], led multiple groups to investigate VEN therapeutic drug monitoring (TDM), yielding heterogeneous results. The single most extensive study, analyzing data from the 2 seminal studies of aza-ven in untreated AML, found no significant exposure-efficacy relationship (in terms of area under the curve-AUC) and a shallow relationship with treatment-emergent neutropenia (neutrophils ≤ 1000/μL) [7]. However, multiple subsequent studies, suggested that: (*i*) there could be a relationship between VEN exposure and response, in terms of trough levels (Cmin) [14,16], stability of Cmin (correlation with the probability to achieve MRD negativity) [8], VEN concentration 6 h post administration, corrected for dose and weight (limited to patients with good or intermediate prognosis [17]); (*ii*) VEN exposure could correlate with neutropenia, in terms of AUC [14,18], Cmin [8] or average concentrations [19]. Moreover, some reports indicate that posaconazole induces VEN accumulation [9,10,17]. As already mentioned, all studies observed a significant interpatient variability [7,8,9,10]; conversely, some studies reported also a significant intrapatient variability [8], while others found intrapatient consistency [9]. All these studies leverage specific pharmacokinetic (PK) analyses, requiring multiple blood samples per day, and do not reflect the real-word practice, with most patients selected for clinical trials and most patients still receiving a 28-day VEN schedule.

The clinical approach at our Institution includes: (*i*) the reduction of the days of VEN administration in patients treated with aza-ven, especially when they are already in response; (*ii*) a careful antifungal prophylaxis, mainly with posaconazole; and (*iii*) a fine tuning of VEN concentrations through TDM if patients are under azoles, within the EMA Summary of Product Characteristics (SmPC, the Prescribing Information equivalent in Europe).

The present study aims to describe the results of our clinical practice, i.e., evaluating the feasibility and the utility of VEN TDM in a real-word setting. In particular, we investigated whether VEN exposure correlated with safety and efficacy, and how posaconazole could impact the management of patients receiving VEN-based treatments.

## 2 Materials and Methods

### 2.1 Study Design and Patients

This observational, monocentric, retrospective and prospective study enrolled patients with AML aged 18 years or older, treated with VEN-containing regimens at Mauriziano Hospital, Torino, Italy, from 06 September 2021 to 13 May 2025, in which VEN had been administered for at least 4 days, for whom at least one VEN plasma concentration had been determined. Patients could have *de novo* or secondary AML, which could be newly diagnosed or R/R. The study aimed to:

i. Evaluate the impact of posaconazole on VEN plasma concentrations in a real-word setting (dose reduction, risk of VEN accumulation, influence on the elimination time). This PK analysis involved the whole study population (PK population), including patients treated with VEN + hypomethylating agents and patients treated with VEN + chemotherapy.
ii. Evaluate whether VEN exposure correlated with treatment safety. In particular, cytopenias (onset and duration), granulocyte colony-stimulating factor (G-CSF) and transfusion requirements, cycle duration (28 days or more), infective and hemorrhagic events were evaluated (any grade). This analysis was limited to patients treated with aza-ven, to avoid obvious bias (safety subset).
iii. Evaluate whether VEN exposure correlated with treatment efficacy. This analysis correlated VEN exposure in the first cycle of aza-ven to the best leukemic response obtained, and it was limited to the first cycle of untreated or R/R patients with >5% of blasts, for whom a bone marrow evaluation was available (efficacy subset); patients treated off-label for MRD eradication were excluded.

Both platelet and red blood cell (RBC) transfusions were expressed in terms of transfusions/day, in order to normalize data for the duration of the cycle. If the patient had 2 or more phases of neutropenia during a single cycle, the total duration was their sum. For more information, please refer to Paragraph S1, protocol details. The subdivision in the different subsets is discussed in Paragraph S2, and more clearly depicted in a flow chart (Fig. S1).

The study was conducted in accordance with the 1964 Helsinki Declaration and approved by the Ethics Committee of Comitato Etico Territoriale (protocol number 0001262 issued on 25 March 2025, approval number CET44). Informed consent was obtained from study patients.

### 2.2 Therapeutic Regimens

Enrolled patients were treated with VEN in association with azacitidine, decitabine or chemotherapy, according to the physicians’ choice. Azacitidine was administered subcutaneously, 75 mg/m^2^/day for 7 consecutive days or in a “5 + 2” scheme; decitabine was administered intravenously, 20 mg/m^2^/die for 5 consecutive days. Chemotherapy regimens included FLAI (fludarabine, high-dose cytarabine, idarubicin), HAM (high-dose cytarabine and mitoxantrone) and MEC (mitoxantrone, etoposide and cytarabine); VEN was associated with chemotherapy in the setting of a hospital-authorized off-label.

VEN was administered orally, once daily, after lunch. The administered dosage was generally 400 mg/day when no antifungal drug was concomitantly prescribed, but it could be reduced per clinical decision. When patients also received antifungal agents, the administered dosage was reduced by at least 75% with posaconazole and voriconazole, and at least 50% with isavuconazole; in these cases, physicians frequently fine-tuned the VEN dosage during the therapeutic cycle or in subsequent cycles based on VEN TDM, always remaining within EMA SmPC (e.g., 100 mg or lower for posaconazole). When VEN was administered with hypomethylating agents, ramp-up was performed in the first cycle, as per EMA SmPC. The duration of VEN administration in patients treated with aza-ven was decided by attending physicians. For further details on VEN scheme modifications in terms of dosage and days of administration compared to VEN SmPC, please refer to the Results section. For additional details on the administration of antifungal agents, please refer to Paragraph S1—protocol details.

### 2.3 Determination of VEN Plasma Concentration

Samples for VEN plasma concentration analysis were collected during routinary morning blood sampling (8:00 a.m.–9:00 a.m.), i.e., approximately 3–4 h before the next VEN ingestion. According to the observational nature of the study, patients underwent VEN concentration analysis as per clinical practice, and no additional hospital visit was required; however, for the purpose of this article, to limit variability, we only considered samples collected at days 5-8-11-15-22-29, +/−1. Moreover, to investigate VEN excretion with and without posaconazole, blood samples were collected at VEN withdrawal and in the subsequent days, depending on clinical follow up visits (up to 10 days after VEN withdrawal).

VEN quantification in plasma was performed using a liquid chromatography coupled to tandem mass spectrometry (LC-MS/MS) method, validated according to the main guidelines on bioanalytical method validation. A comprehensive description of the methodology employed is available in the Supplementary Materials (Paragraph S3, VEN plasma concentration analysis).

### 2.4 Data Analysis

VEN plasma concentrations were correlated to the administered dosage and to patient characteristics (age, sex, body mass index-BMI, body surface-BSA), both in the whole population, in the presence of posaconazole (Pos group) and in the absence of any antifungal agent (No-AA group). Further confounding factors (kidney or hepatic failure, other interfering drugs) were considered. VEN concentrations were compared at different timepoints (to verify its accumulation) and after withdrawal (to investigate its excretion), in the Pos group vs. the No-AA group. VEN exposure was then correlated to safety and efficacy, and it was defined through the following parameters: (1) the highest concentration available for each patient in each cycle; as our determinations are very similar to trough levels (Cmin), we called this parameter hCmin, (2) the average of VEN plasma concentrations in each cycle (aCmin), (3) the days of VEN administration, and (4) the exposure index, i.e., the product of aCmin for the days of VEN administration.

From a technical standpoint, we used Python version 3.13 as our primary analytical tool, leveraging widely used statistical libraries, including Pandas for data manipulation, SciPy and statsmodels for statistical analyses, and Matplotlib and Seaborn for data visualization. To assess data normality, Shapiro-Wilk tests were performed. Point-Biserial Correlation, Spearman Correlation, the Mann-Whitney U test, logistic regression, and least square regression were applied as part of our analyses. Additionally, to account for repeated measurements within patients, multivariate analysis was performed using linear mixed-effects models with patient-specific random intercepts. A significance level of 0.05 was adopted for all statistical tests.

## 3 Results

### 3.1 Patient Characteristics

Patient characteristics are detailed in Table 1. Briefly, the overall study population, utilized for PK analysis, included 43 patients, corresponding to 163 cycles: 141 (86.5%) with VEN and azacitidine, 9 (5.5%) with VEN and decitabine, and 13 (8%) with VEN and chemotherapy. Most patients received several cycles, with or without posaconazole prophylaxis, and sometimes at different dosages of VEN (please refer to Section 3.2). Six patients (13.9%) received more than one type of VEN-based regimen. The median age was 70.6 years, with a significant male prevalence. No patient had moderate or severe liver impairment, and only 1 patient had creatinine clearance < 30 mL/min/1.73 mq. Eastern Cooperative Oncology Group (ECOG) performance status scores at diagnosis were ≥2 in 23 patients (53.5%), reflecting the strong real-life nature of the study. The use of VEN-interfering drugs other than azoles was limited to few cases of deferasirox (4 cycles) and isoniazid (3 cycles), which are known to moderately reduce and increase, respectively, VEN concentrations. Conversely, the use of antifungal medications, particularly posaconazole, was extremely common (>40%).

The safety subset included all patients treated with aza-ven (35 patients, 141 cycles), with a median age of 71.2 years and ECOG at diagnosis ≥ 2 in 19 patients (54.2%). In the PK population and in the safety subset, 60.1% and 65.2% of the analyzed cycles were for consolidation/maintenance, respectively.

The efficacy subset included 29 patients (corresponding to 29 cycles, as only the first cycle of aza-ven patients with active disease was considered), with a median age of 73.2 and ECOG ≥ 2 in 15 patients (51.7%). There were 23 untreated (79.3%) and 6 R/R (20.7%) patients; 16 (55.2%) had secondary AML, and 3 (10.3%) had therapy-related AML. According to the 2022 European Leukemia Net (ELN) risk stratification [20], 13.8% had favorable, 31% had intermediate, and 55.2% had adverse risk AML; according to the 2024 ELN stratification [21] for less intensive therapies, 13.8% had favorable, 62.1% had intermediate, and 24.1% had adverse risk AML.

**Table 1:** Demographic and clinical characteristics of the study population (overall PK population, safety, and efficacy subsets).

Characteristics		Overall Population (PK Population)		Safety Subset		Efficacy Subset
		Pts (43)	Cycles (163)	Pts (35)	Cycles (141)	Pts (29)
**Sex Male/Female, n (%)**		29/14 (67.4/32.6%)	119/44 (73.0%/27.0%)	23/12 (65.7%/34.3%)	105/36 (74.5%/25.5%)	21/8 (72.4%/27.6%)
**Age (mean ± SD)**		70.6 ± 9.6	73.8 ± 8.3	71.2 ± 7.1	74.9 ± 7.4	73.2 ± 7
**BMI (mean ± SD)**		25.3 ± 3.6	25 ± 2.8	25.1 ± 3.4	24.9 ± 2.6	25.7 ± 3.1
**BSA (mean ± SD)**		1.8 ± 0.2	1.8 ± 0.2	1.77 ± 0.2	1.82 ± 0.15	1.8 ± 0.2
**Hepatopathy (Child Pugh B or C), n (%)**		0 (0.0%)	0 (0.0%)	0 (0.0%)	0 (0.0%)	0 (0.0%)
**eGFR < 30 mL/min/1.73 mq, n (%)**		1 (2.3%)	2 (1.2%)	1 (2.9%)	2 (1.4%)	0 (0.0%)
**ECOG, n (%)**	**0–1**	20 (46.5%)	87 (53.4%)	16 (45.7%)	72 (51.1%)	14 (48.3%)
	**2**	16 (37.2%)	58 (35.6%)	13 (37.1%)	53 (37.6%)	10 (34.5%)
	**>2**	7 (16.3%)	18 (11.0%)	6 (17.1%)	16 (11.3%)	5 (17.2%)
**Concomitant CYP3A4 modulators, n (%)**		NA*	86 (52.8%)	NA*	67 (47.5%)	19 (65.5%)
** Posaconazole, n (%)**		NA*	66 (40.5%)	NA*	52 (36.9%)	17 (58.6%)
** Vorico/isavuconazole, n (%)**		NA*	13 (8.0%)	NA*	9 (6.4%)	1 (3.5%)
** Deferasirox, n (%)**		NA*	4 (2.4%)	NA*	3 (2.1%)	0 (0.0%)
** Isoniazid, n (%)**		NA*	3 (1.8%)	NA*	3 (2.1%)	1 (3.5%)
**Therapies, n (%)**						
**Aza-ven**		35 (81.4%)	141 (86.5%)	35 (100.0%)	141 (100.0%)	29 (100.0%)
**Deci-ven**		4 (9.3%)	9 (5.5%)	0 (0.0%)	0 (0.0%)	0 (0.0%)
**CHT-ven**		11 (25.6%)	13 (8.0%)	0 (0.0%)	0 (0.0%)	0 (0.0%)
**>1 type of ven-based treatment**		6 (13.9%)	NA	NA	NA	NA
**Disease, n (%)**						
***De novo* vs. secondary AML**		19/24 (44.2%/55.8%)	98/65 (60.1%/39.9%)	16/19 (45.7%/54.3%)	91/50 (64.5%/35.5%)	13/16 (44.8%/55.2%)
**Therapy related**		4 (9.3%)	6 (3.7%)	4 (11.4%)	5 (3.5%)	3 (10.3%)
**AML status**						
** Untreated**		NA#	45 (27.6%)	NA#	38 (27.0%)	23 (79.3%)
** Relapsed/Refractory**		NA#	20 (12.3%)	NA#	11 (7.8%)	6 (20.7%)
** Consolidation/maintenance**		NA#	98 (60.1%)	NA#	92 (65.2%)	0 (0.0%)
**ELN risk stratification 2022**						
** Favorable**						4/29 (13.8%)
** Intermediate**						9/29 (31.0%)
** Adverse**						16/29 (55.2%)
**ELN risk stratification 2024**						
** Favorable**						4/29 (13.8%)
** Intermediate**						18/29 (62.1%)
** Adverse**						7/29 (24.1%)

Notes: Pts: patients. BMI: Body Mass Index. BSA: Body Surface Area. eGFR: Estimated Glomerular Filtration Rate. ECOG: Eastern Cooperative Oncology Group. Vorico: voriconazole. Aza-ven: azacitidine-venetoclax. Deci-ven: decitabine-venetoclax. CHT-ven: chemotherapy-venetoclax. AML: Acute Myeloid Leukemia. ELN: European Leukemia Net. NA: not applicable. *: NA, as the same patient could undergo some cycles with and some cycles without interfering drugs. #: NA, as the same patient typically undergoes the first treatment as untreated or R/R, and then proceeds to consolidation.

### 3.2 VEN Plasma Concentrations

VEN plasma concentrations were evaluated in 43 patients (PK population), with a total number of 290 VEN determinations over 163 cycles (induction and consolidation/maintenance) at specific timepoints (days 5-8-11-15-22-29), as described in Table 2.

**Table 2:** Distribution of Venetoclax determinations along cycles and timepoints; number of cycles requiring dose modifications for Therapeutic Drug Monitoring (TDM).

Total N° of VEN Determinations	
PK population	290
Safety subset	247
Efficacy subset	64
**N° of VEN Determinations Per Cycle (PK Population)**	
1	82
2	49
3	22
4	7
5	2
6	1
**N° of VEN Determinations Per Timepoint (PK Population)**	
Day 5	96
Day 8	111
Day 11	33
Day 15	40
Day 22	9
Day 29	1
**Days of VEN Administration, Mean (SD)**	
PK population	11.64 (4.54) days
Safety subset	11.47 (3.96) days
Efficacy subset	12.28 (5.11) days
**VEN Dose Modifications, n (%)**	
Cycles with VEN dose modifications due to TDM/evaluable cycles* (PK population)	27/156 (17.3%)
When posaconazole was co-administered	22/65 (33.8%)
When voriconazole was co-administered	1/12 (8.3%)
Without the co-administration of antifungal agents	4/79 (5.1%)

Notes: VEN: venetoclax. PK: pharmacokinetic. SD: standard deviation. *dose modifications due to other causes were excluded from this analysis.

**Figure 1:**
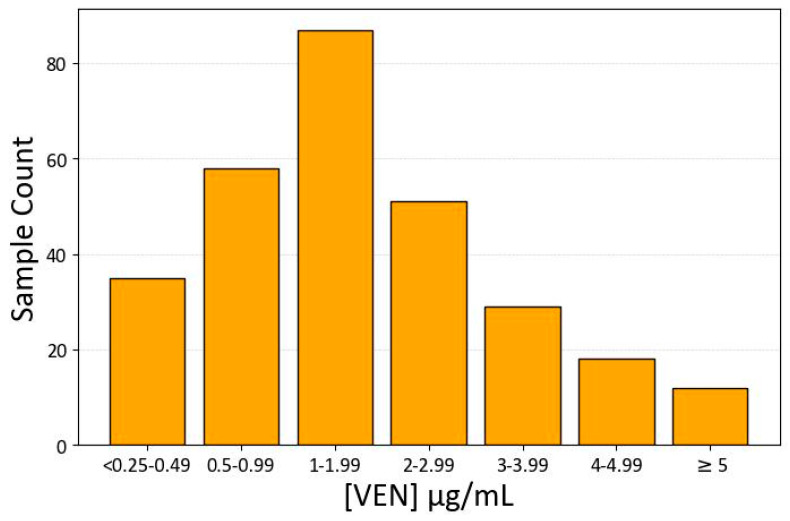
**Distribution of VEN determinations per concentration intervals.** [VEN]: venetoclax plasma concentration.

The range of VEN plasma concentrations was extremely wide (<0.25 to 9.3 μg/mL), as shown in Fig. 1. There were 35 (12.4%) very low values (<0.25–0.49 μg/mL): most them (30/35) were associated with reduced dosages of VEN per physician’s choice (“intentional” underdose: this occurred especially in older, fragile patients in consolidation/maintenance; accordingly, the median age of patients with these 30 determinations was 81.34 years). However, 5 determinations were associated with standard dosages (“unintentional” underdose): 400 mg in 2 cases, 100 mg with posaconazole in 2 cases and 50 mg with posaconazole in 1 case. On the other side, there were 29 (10%) very high values (>4 μg/mL): in 25 of them, posaconazole was administered with VEN (100 mg in 10 cases, 70 mg in 9, and 50 mg in 6), while in 4 of them VEN was administered without antifungal agents. In terms of patients (instead of VEN determinations), “unintentional” underdose occurred in 4 patients (9.3% of the PK population), while overdose occurred in 16 patients (37.2% of the PK population).

These data are mitigated by the policy at our Institution, where physicians are used to fine-tune VEN dosage within VEN SmPC when patients are under azoles, based on TDM. The dosage of administered VEN was modified based on TDM in 27 of 156 evaluable cycles (7 cases were deemed not evaluable as modifications were due to other reasons, such as infections). Of these, posaconazole had been co-administered in 22 cases (81.5%) and voriconazole in 1 (3.7%); in 4 cases (14.8%), dose modifications occurred in the absence of antifungal agents. These 4 cases correspond to the overdoses described above, and physicians decided to modify VEN dosage based on the emerging scientific literature, although outside VEN SmPC. The use of deferasirox and isoniazid, in our series, did not seem to significantly impact VEN concentrations: these drugs were utilized in 6 cycles, 2 with concomitant posaconazole administration and 4 without. There were 2 dose modifications due to TDM, both when posaconazole was co-administered.

Going into detail, when posaconazole was co-administered, VEN was modified in 22 over 65 evaluable cases (33.8%); considering only cycles when posaconazole was given for the first time, the rate was 11/23 (47.8%). Dose modifications occurred both for underdose and overdose, at every dosage (50, 70 or 100 mg), as detailed in Table 3 (at 100 mg of VEN, in case of underdose, dose was not furtherly increased, as against EMA SmPC). One patient assumed 200 mg of VEN with posaconazole by mistake.

**Table 3:** Details on VEN dose modification when posaconazole was co-administered.

Starting VEN Dosage When Posaconazole Was Co-Administered	Frequency, n (%)	Dose Modifications for TDM, n (%)	Dose Modifications for Underdose, n (%)	Dose Modifications for Overdose, n (%)
50 mg	18 (27.7%)	5/18 (27.8%)	4/5 (80%)	1/5 (20%)
70 mg	18 (27.7%)	7/18 (38.9%)	2/7 (28.6%)	5/7 (71.4%)
100 mg	28 (43.1%)	10/28 (35.7%)	NA#	10/10 (100%)
200 mg †	1 (1.5%)	0/1 (0%)	NA	NA
Total	65 (100%)*	22/65 (33.8%)	6/22 (27.3%)	16/22 (72.7%)

Notes: VEN: venetoclax. TDM: Therapeutic Drug Monitoring. NA: not applicable. #: the dosage of VEN could not be increased even in the presence of underdose, as per EMA Prescribing information. †: one patient assumed 200 mg of VEN with posaconazole by mistake. *65 cycles were deemed evaluable, as cycles with VEN dose modifications due to other causes than TDM were excluded from this analysis.

Trying to dissect this extreme variability, we investigated the influence of patients’ characteristics on VEN plasma concentrations in a multivariate analysis (Table S1), but found no significant correlation with gender, age, BMI, BSA or VEN-associated treatment (azacitidine, decitabine or chemotherapy). The only analyzed variable that influenced VEN plasma concentration was the administered dose of VEN, both in the Pos group and in the No-AA group.

Finally, as blood samples for the determination of VEN concentrations were withdrawn 3 to 4 h before the next dose, we performed a limited analysis on the impact of this delay. As shown in Fig. S2, the impact seems extremely modest, ranging from 0 to 15%, at least for VEN concentrations < 2.3 μg/mL.

### 3.3 Role of Posaconazole in VEN Accumulation

**Figure 2:**
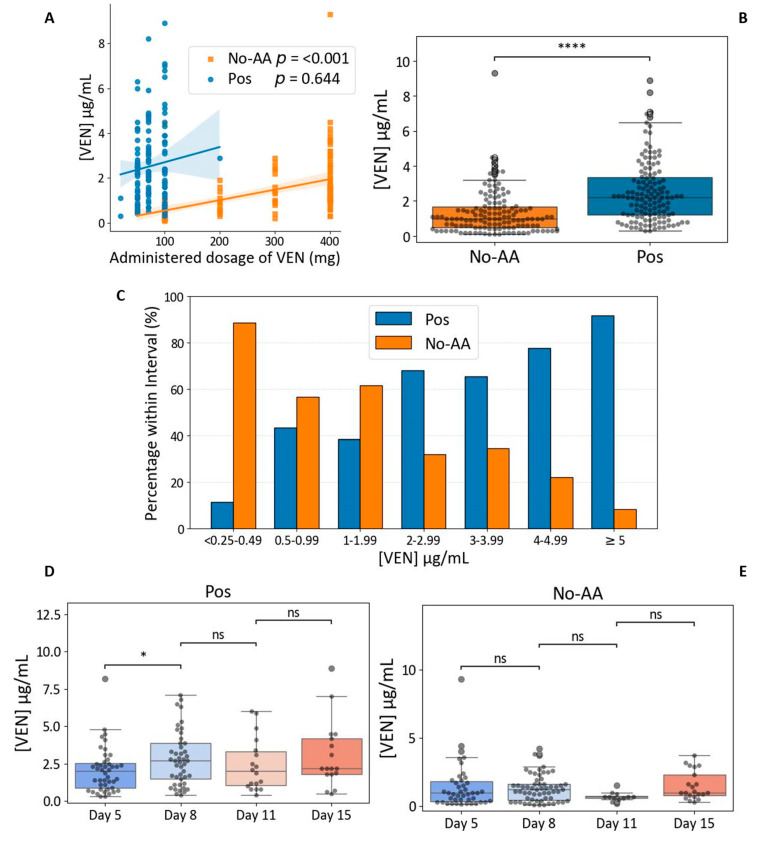
**Posaconazole induces venetoclax accumulation.** All analyses compare venetoclax (VEN) plasma concentrations obtained during posaconazole co-administration (Pos) with those obtained in the absence of any antifungal agent (No-AA). (**A**) Correlation between the administered VEN dosage and VEN plasma concentrations (Pos vs. No-AA). One patient assumed 200 mg of VEN with posaconazole by mistake. (**B**) Overall distribution of VEN plasma concentrations (Pos vs. No-AA). (**C**) Rate of Pos vs. No-AA per VEN concentration interval (each interval represents 100%). (**D**,**E**) Distribution of VEN plasma concentrations per timepoint (Pos (**D**) vs. No-AA (**E**)): VEN accumulation is evident in the Pos group from day 5 to day 8. For plots D and E, a linear mixed-effects model with random subject-specific intercepts and with the administered dose as covariate was used. Wald’s tests were used to assess differences between timepoints. [VEN]: venetoclax plasma concentration; *p < 0.05, ****p < 0.0001, ns not significant.

Given results in 3.2, we decided to investigate in detail the impact of posaconazole on VEN plasma concentrations. First, we used standard univariate analysis to quantify the correlation between the administered dose of VEN and plasma concentrations (Fig. 2A). Apparently, there was no correlation in the Pos group (*p* = 0.64), while it was extremely clear in the No-AA group (*p* < 0.001). This result was not confirmed in the multivariate analysis (Table S1) performed by using linear mixed effect models with subject-specific random intercepts, in order to account for the fact that several measurements are not independent, as they come from the same patient. In fact, the multivariate analysis showed a significant correlation for both groups of patients, as described in the previous paragraph. Taken together, these findings indicate that for each subject of the Pos group an increase or a decrease of the administered dosage has an impact on VEN plasma concentration (multivariate analysis); however, the correlation between dosage and VEN plasma concentrations across the group is weak, reflecting substantial inter-individual variability (Fig. 2A).

The coadministration of posaconazole was generally associated with higher VEN concentrations, as shown in Fig. 2B (*p* < 0.0001) and Fig. S3. Extremely high VEN concentrations (>5 μg/mL) were almost exclusively found in this population, but patients assuming posaconazole could also have very low VEN concentrations (Fig. 2C).

We next investigated whether posaconazole, by slowing down VEN metabolism, could cause VEN accumulation during the cycle. We compared VEN concentrations at different timepoints, both in the No-AA and in the Pos groups (days 22 and 29 were excluded for the limited sample size). As shown in Fig. 2D, VEN concentrations on day 8 are significantly higher compared to day 5 in the Pos group (*p* = 0.03); when we compared only patients with available VEN concentrations both on day 5 and on day 8, the phenomenon was even more clear (*p* = 0.0013, Fig. S4). Afterwards, on day 11, there is a trend toward a reduction in VEN concentrations, likely due to the VEN fine-tuning policy at our Institution, that prevented further accumulation over time. Notably, the same analyses in the No-AA group are characterized by a totally flat trend, suggesting that VEN has already reached the steady state by day 5 and does not undergo significant accumulation (Fig. 2E, Figs. S3 and S4).

Finally, we investigated whether posaconazole could prolong VEN exposure after the end of treatment, by slowing down its metabolism. Since most of the patients were outpatients, we could collect close-up VEN concentrations after the end of treatment only in a minority of them. VEN concentrations at withdrawal and at least 2 further VEN concentrations after the end of treatment were available in 7 cycles, 6 with concomitant administration of posaconazole and one without antifungal agents. In the latter one, VEN half-life (T/2) resulted 18.8 h, in line with literature data. The same patient also underwent a subsequent aza-ven cycle with posaconazole, and VEN T/2 resulted 32.9 h (Fig. S5). In the other 5 evaluable cycles with posaconazole, all from different patients, VEN T/2 ranged from 24.9 h to 59.9 h. Longer T/2 were found in patients with higher VEN concentrations at withdrawal, suggesting that a slower metabolism of the drug leads to its accumulation (Table S2). While a meticulous PK analysis was outside the purpose of the present study, the net consequence of this phenomenon is that we could still find measurable VEN plasma concentrations after more than 5 days from withdrawal in 4 patients treated with posaconazole (Table S3). In summary posaconazole, particularly in some patients, can cause an increase in VEN concentrations, VEN accumulation over time and a delayed excretion, significantly increasing the patient exposure to the drug.

### 3.4 Exposure-Safety Analysis

In order to evaluate the role of VEN TDM in clinical practice, we investigated the correlation between safety and exposure to VEN in the cohort of patients treated with aza-ven (safety subset). Exposure to VEN was analyzed in terms of the highest and average Cmin (hCmin and aCmin), days of VEN administration, exposure index, and Cmin stability (VEN concentrations always within the 0.5–4 μg/mL range). Safety was evaluated through multiple parameters, as detailed in Table 4, Figs. S6–S17 and Tables S4 and S5.

Exposure to VEN was clearly correlated to multiple safety items; the two most informative parameters were hCmin and the exposure index, which positively correlated with: (*i*) the development and the duration of both profound (N < 100/μL) and severe (N < 500/μL) neutropenia; (*ii*) G-CSF requirement; (*iii*) the need for platelet transfusions; (*iv*) cycle duration, i.e., the need to postpone the next cycle; (*v*) infective adverse events (for details on correlation significance, please refer to Table 4). The same trends could be observed with aCmin, but with inferior statistical significance and correlation coefficient, likely suggesting that: (*i*) high VEN concentrations, even if transient, can be detrimental for the medullary function, and (*ii*) higher VEN concentrations are associated with delayed drug elimination, thus prolonging VEN exposure. The duration of VEN administration significantly correlated only with the development of both profound and severe neutropenia. However, the exposure index, which is the product of aCmin by the days of VEN administration, was more informative than aCmin in almost every considered item: overall, this suggests that both VEN concentrations and the days of treatment concur to medullary toxicity. Bleeding events and the need for red blood cell transfusions did not correlate with any exposure parameter.

**Table 4:** Correlation between VEN exposure and multiple safety parameters.

Safety Parameters	hCmin (Coeff., *p*-Value)	aCmin (Coeff., p-Value)	Days of VEN Adm (Coeff., *p*-Value)	Exposure Index (Coeff., *p*-Value)
**N < 100/μL**	0.228 (0.008)	0.202 (0.018)	0.181 (0.034)	0.226 (0.008)
**Duration of N < 100/μL**	0.4 (0.0130)	0.356 (0.028)	0.063 (0.706)	0.38 (0.018)
**N < 500/μL**	0.177 (0.039)	0.155 (0.071)	0.255 (0.003)	0.234 (0.006)
**Duration of N < 500/μL**	0.234 (0.035)	0.181 (0.105)	0.114 (0.312)	0.21 (0.060)
**G-CSF admin/cycle**	0.37 (<0.001)	0.384 (<0.001)	0.117 (0.176)	0.346 (<0.001)
**Plts < 25.000/μL**	0.152 (0.077)	0.104 (0.228)	0.106 (0.216)	0.07 (0.419)
**Plt transfusions/day**	0.206 (0.016)	0.153 (0.075)	0.064 (0.454)	0.158 (0.067)
**RBC transfusions/day**	0.133 (0.122)	0.099 (0.251)	−0.108 (0.21)	0.052 (0.549)
**Total cycle duration**	0.305 (<0.001)	0.264 (0.003)	0.131 (0.139)	0.276 (0.002)
**Infective AEs**	0.161 (0.062)	0.157 (0.068)	0.143 (0.096)	0.177 (0.040)
**Bleeding AEs**	−0.031 (0.724)	−0.029 (0.741)	−0.101 (0.248)	−0.092 (0.295)

Notes: hCmin and aCmin: highest and average venetoclax plasma concentration for each patient in each cycle. Days of VEN adm: days of venetoclax administration. Coeff: correlation coefficient (Point biserial for binary variables, Spearman’s for continuous variables). N: neutrophils. G-CSF: granulocyte colony-stimulating factor. Plt: platelets. RBC: red blood cells. AE: adverse event. NonR: non responders. ORR: overall response rate, including CR (complete response), CRi (CR with incomplete hematologic recovery) and PR (partial response). cCR: composite response, including CR and CRi. The duration of neutropenia was calculated only in patients actually undergoing N < 100 or 500/mmc (i.e., duration of neutropenia > 0). Highlighted values are statistically significant (*p* < 0.05); underlined values are borderline for statistical significance (*p* < 0.07).

Starting from the hypothesis that even one single episode of overdose could have an impact on the medullary function, we compared the safety outcomes in cycles where VEN concentrations always remained in the 0.5–4 μg/mL range vs. cycles with at least one value >4 μg/mL vs. cycles with at least one value <0.5 μg/mL. Results are shown in Tables S4 and S5 and Fig. S17: it is extremely clear that if patients remain in that range (or below) experience less toxicity in terms of cytopenia compared to patients undergoing even a single VEN concentration above that range.

### 3.5 Exposure-Efficacy Analysis

We next investigated whether VEN exposure correlated with its antileukemic activity in the efficacy subset. Response was evaluated in terms of CR (complete remission), CRi (CR with incomplete hematologic recovery according to ELN 2022 criteria), cCR (composite response, including CR and CRi), PR (partial response according to ELN 2022 criteria) and ORR (overall response rate, including CR, CRi and PR). The response rate of this cohort is detailed in Table S6. Briefly, ORR and cCR of the entire efficacy subset were 69% and 55%, respectively. This is in line with the literature, as: (*i*) our cohort included R/R patients; (*ii*) our cohort was enriched in secondary AML, therapy-related AML and, above all, intermediate/adverse risk AML according to both ELN 2022 and ELN 2024 criteria (Table 1); (*iii*) 51.7% of the patients had ECOG ≥ 2. Accordingly, when response was stratified according to the ELN 2022 and 2024 risk classification, cCR were 100% and 75% in the favorable group, 78% and 61% in the intermediate group, and 31 and 29% in the adverse risk group, respectively (Table S6).

VEN exposure was again expressed in terms of hCmin, aCmin, days of VEN administration and exposure index. No statistically significant correlation was found between VEN exposure (any item) and response (both ORR and cCR), as shown in Table 5, Figs. S18 and S19.

**Table 5:** Correlation between VEN exposure and response.

Efficacy Parameters	hCmin (Coeff., *p*-Value)	aCmin (Coeff., *p*-Value)	Days of VEN Adm (Coeff., *p*-Value)	Exposure Index (Coeff., *p*-Value)
**NonR vs. ORR**	−0.1 (0.60)	−0.02 (0.92)	−0.21 (0.27)	−0.09 (0.66)
**NonR vs. cCR (CR, CRi)**	−0.04 (0.84)	0.02 (0.91)	−0.13 (0.53)	−0.0 (>0.99)

Notes: hCmin and aCmin: highest and average venetoclax plasma concentration for each patient in each cycle. Days of VEN adm: days of venetoclax administration. Coeff: correlation coefficient (Point biserial). NonR: non responders. ORR: overall response rate, including CR (complete response), CRi (CR with incomplete hematologic recovery) and PR (partial response). cCR: composite response, including CR and CRi.

## 4 Discussion

Managing elderly patients with AML is extremely challenging: prolonging their OS is as important as improving their quality of life; being active against the disease is as important as reducing treatment side effects, hospital visits and admissions. In other words, it is essential to minimize treatment to the least possible to be active against the disease but avoid side effects in this unfit population. The recent introduction of aza-ven had a major impact on this population, bringing both more responses and more toxicity, that the scientific community is trying to mitigate with at least 2 complementary approaches: (*i*) reducing the days and/or the dosage of VEN administration [3,4,5,6], and (*ii*) investigating whether VEN TDM could help finding the optimal concentration range to maximize efficacy and minimize side effects, in the context of a drug characterized by a high interpatient variability. VEN TDM is currently being studied in several clinical trials, which led to controversial results so far; moreover, these trials often rely on the classical aza-ven schedule (28 days, 400 mg or equivalent), and do not reflect the real-word experience, where it is impossible to collect multiple blood samples at specific hours as in PK studies, and VEN concentrations can be determined only during clinical visits.

In the present study we investigated the feasibility and utility of VEN TDM in the real-word setting of our Institution, where the days of VEN administration are generally reduced (typically <14 days), the treated population is less fit compared to the one in clinical trials and enriched in high-risk patients, with the consequent need to frequently use posaconazole. The real-world nature of the study did not allow a classical PK analysis: blood samples were collected at specific timepoints (days 5, 8, 11, 15, 22, and 29) when they matched clinical visits, so that each patient could have from 1 to 6 VEN determinations per cycle; moreover, they were collected during the morning withdrawal, while VEN was administered at lunch, i.e., 3–4 h later. The impact of this delay seems negligible, according to our data, at least up to VEN concentrations < 2.3 μg/mL, in line with time-concentrations curves from other PK studies [22], that show a substantial flat trend in the last hours before the next dose; however, it is possible that with higher VEN concentrations the difference could be more remarkable, as the same curves seem to suggest. Our VEN determinations, therefore, cannot be compared exactly to Cmin from other studies, as they could be slightly overestimated, mainly for high and over-the-range values.

Overall, however, our data were in line with the existing literature. First, we observed that the range of VEN concentrations was extremely wide, confirming previous reports [8,14,18,23]. It was even wider and poorly predictable when posaconazole was co-administered, due to an extremely high interpatient variability, so that the same dosage could lead to a <0.5 or >6 μg/mL concentration. There are no validated thresholds for VEN plasma concentrations. However, the majority of patients in clinical trials that led to FDA approval of VEN had Cmin between 0.5 and 4 μg/mL [11,22,24]. Accordingly, Wang et al. chose this range to perform dose adjustments [8], showing that patients with Cmin consistently within this range had higher probability to achieve MRD negativity. In the clinical practice at our Institution, patients with antifungal co-administration whose VEN plasma concentrations were outside the 0.5–4 range, or very near these thresholds, underwent dose adjustments within VEN EMA SmPC (e.g., 100 mg or less with posaconazole). In patients not assuming azoles, dose adjustments were seldom decided by the attending physicians based on the emerging scientific literature, although outside of VEN SmPC.

Unintentional outliers were significantly more frequent in case of posaconazole co-administration, with overdose more frequent than underdose. Accordingly, dose adjustments were remarkable in this population (33.85% of cycles in the Pos group). Adjustments occurred in 47.8% of cycles where VEN-posaconazole were co-administered for the first time vs. 26.19% in subsequent cycles, indicating a substantial intrapatient consistency and the fact that an accurate VEN fine-tuning during the first cycle is often able to identify the best VEN dosage, that is generally maintained in subsequent cycles. Outliers (and dose adjustments) occurred with every starting dose (50, 70 or 100 mg), but they were more common with 70 and 100 mg, especially for overdose. The fact that posaconazole slows down VEN metabolism, sometimes inducing overdose, is well known, but here we showed two further practical consequences: (*i*) the risk of accumulation, i.e., the increase of VEN concentrations along cycles, in accordance with [10], and (*ii*) a delayed drug clearance, leading to active concentrations of venetoclax far beyond the day of drug withdrawal, further increasing VEN exposure. These results are in line with findings from PK analyses by Agarwal et al. [11], and could lead to the conclusion that 50 mg is the best starting dose. However, the accumulation phenomenon does not occur in all posaconazole-treated patients, with some patients undergoing underdose even with the 100 mg administration, in a totally unpredictable way: patients with reduced/unknown compliance were excluded from the analysis, and we found no strong correlation with any patient characteristics (age, gender, BMI or BSA). This variability is likely the result of multiple factors, such as fat food intake, renal and liver function, plasma proteins, and probably CYP3A4 polymorphisms (even though a specific polymorphism has not been found yet [14]). In the Pos group, it could also be the result of posaconazole accumulation [8], as this drug is also characterized by considerable variability. In summary, we showed that VEN concentrations cannot be inferred based on clinical characteristics and drug dosage, especially in the posaconazole population, but can only be determined directly by TDM.

Subsequently, we investigated whether VEN TDM performed in a real-word setting had an impact on clinical outcomes; this analysis was restricted to patients treated with aza-ven. More in particular, we wanted to assess whether singular high VEN concentrations (hCmin), the average VEN concentration during the cycle, the duration of VEN treatment, or a global measure of VEN exposure (which combined both VEN concentrations and days of administrations) had clinical consequences. In order to assess the latter item, we created the “exposure index”, i.e., the product of the average VEN plasma concentrations in each cycle by the days of VEN administration. The exposure index is basically a simplification of the total AUC: it is represented by a rectangle in the time-concentration curve, while the AUC is the integral, calculated with the trapezoidal rule. This choice was made as: (*i*) an accurate PK analysis was unfeasible in a real-world setting, so that our estimated total AUC would have been extremely approximated, with limited significance or reproducibility and (*ii*) we wanted to create a parameter that could be easily used in the real-world setting, even when the cycle duration is not homogeneous. In terms of safety, we showed that both hCmin and the exposure index had a strong correlation with medullary toxicity and its consequences, including the onset and the duration of neutropenia, infective adverse events, the need for platelet transfusion, G-CSF administration, and cycle delay. Accordingly, multiple safety outcomes in cycles with all VEN concentrations within the 0.5–4 μg/mL range were significantly better than those with at least one value above this range, indicating that also singular VEN peaks could affect the medullary function (Fig. S17 and Table S5). The duration of VEN administration alone was the least informative parameter, correlating only with neutropenia onset; however, the average duration of VEN administration in the safety cohort was 11.47 days (standard deviation 3.96), due to the policy of our Centre: cycles with longer VEN administration (>14 days) are poorly represented in our study population, probably impacting on statistical analyses (e.g., in Fig. S9, panel C, the few cycles with >20 days of VEN administration are all associated with >10 days of severe neutropenia, even though this correlation is not statistically significant). Overall, our study shows that both VEN concentrations (especially in terms of hCmin) and the duration of VEN treatment likely concur to medullary toxicity.

Finally, we investigated whether VEN exposure correlated with response in patients treated with aza-ven, but no statistically significant correlation was found. This is apparently in contrast with findings by Wang et al. and Kobayashi et al. [8,14]; however, in both studies, non-responding patients had very low VEN concentrations; in [14], the average Cmin in non-responders was 0.587 μg/mL vs. 0.980 μg/mL in responders. In our population, mean aCmin in non-responders and responders (cCR) were 1.93 μg/mL and 1.99 μg/mL, respectively; moreover, just 1 patient had aCmin of 0.4 μg/mL (in the non-responder group). Our study cannot exclude, hence, that very low VEN concentrations could correlate with efficacy, as suggested by [8,14].

This study has several limitations. First, it is an observational study; VEN dose adjustments were performed at the discretion of attending physicians, with no fixed thresholds nor recommended dose variations. Since these adjustments were performed within VEN SmPC when antifungal agents were co-administered, and outside the SmPC when VEN was administered alone, the rate of dose adjustments between the posaconazole and the No-AA groups should not be directly compared, as physicians could have had different approaches. More in general, without a standardized protocol and a randomization, it is impossible to demonstrate the benefit of VEN dose modifications according to TDM. Second, the sample size is relatively limited, especially for: (*i*) the efficacy analysis (29 patients), (*ii*) the posaconazole dose-stratified analysis (50 vs. 70 vs. 100 mg), and (*iii*) cycles with VEN administered for >14 days (15/141 cycles in the safety subset); this could have affected the statistical power and may limit the generalizability of our results. Third, as discussed above, our Cmin could be slightly overestimated compared to values found in the literature, mainly for intermediate-high values, and should not be directly compared. Finally, we had very few posaconazole concentrations, and we could not verify whether its accumulation could be associated with VEN accumulation, as it has already been postulated [8], and our preliminary data suggests.

## 5 Conclusions

This real-world study supports the feasibility of VEN TDM in a real-world setting and suggests that it may be associated with improved safety management, particularly when posaconazole is co-administered. However, the optimal PK parameter and its minimum and maximum thresholds, as well as the best dose-titration strategy, have to be determined, and the clinical benefit of such dose adjustments confirmed, in prospective interventional studies.

## Data Availability

The data that support the findings of this study are available from the Corresponding Author, Valentina Gaidano, upon reasonable request.

## Supplementary Materials

The supplementary material is available online at https://www.techscience.com/doi/10.32604/or.2026.078245/s1.
