# Supplementary material for: Real-World Experience with Venetoclax Therapeutic Drug Monitoring in Acute Myeloid Leukemia: Role of Posaconazole, Correlation with Safety and Efficacy
Source: Oncol Res. 2026 Jul 16;34(8):12. doi: 10.32604/or.2026.078245 (PMC13397345; doi:10.32604/or.2026.078245)
Supplement: Supplementary file 1 [file OncolRes-34-78245-s001.zip › TSP_OR_78245-s001.pdf]

Supporting Information

Supplemental Materials and Methods

Paragraph S1. Protocol details

Paragraph S2. Overall population, safety, and efficacy subsets

Paragraph S3. VEN plasma concentration analysis

Supplemental Tables

Table S1. Effect of patient characteristics on VEN plasma concentration (Multivariate analysis)

Table S2. Calculated VEN T/2 in patients assuming posaconazole

Table S3. VEN plasma concentrations after multiple days from withdrawal, in the Pos group

Table S4. Proportion of cycles with VEN concentrations always within the 0.5-4 μg/mL range vs cycles with out-of range value

Table S5. Comparison of safety outcomes in cycles with VEN concentrations within the 0.5-4 μg/mL range *vs* cycles with at least one value above or below

Table S6. Response rates in the whole efficacy subset and in specific subgroups

Supplemental Figures

Figure S1. Flow chart illustrating the whole study population and the different subsets

Figure S2. Comparison between approximated Cmin and true Cmin

Figure S3. Comparison between Ven determinations at days 5, 8, 11 and 15 in the Pos group vs the No-AA group

Figure S4. VEN accumulation between Day5 and Day8 in patients with both determinations available (paired data)

Figure S5. Comparison between VEN decrease after withdrawal in the presence and in the absence of posaconazole

Figure S6. Correlation between VEN exposure and profound neutropenia (N< 100/μL)

Figure S7. Correlation between VEN exposure and duration of profound neutropenia (days with N< 100/μL)

Figure S8. Correlation between VEN exposure and severe neutropenia (N<500/μL).

Figure S9. Correlation between VEN exposure and duration of severe neutropenia (days with N<500/μL)

Figure S10. Correlation between VEN exposure and G-CSF requirement during each cycle

Figure S11. Correlation between VEN exposure and grade 4 thrombocytopenia (platelets <25.000/ μL

Figure S12. Correlation between VEN exposure and platelet transfusion requirements.

Figure S13. Correlation between VEN exposure and RBC transfusion requirements

Figure S14. Correlation between VEN exposure and total cycle duration

Figure S15. Correlation between VEN exposure and infective adverse events

Figure S16. Correlation between VEN exposure and bleeding adverse events

Figure S17. Comparison of multiple safety outcomes in cycles with VEN concentrations within the 0.5-4 µg/ml range *vs* cycles with at least one value above or below

Figure S18. Correlation between VEN exposure and overall response rate (ORR)

Figure S19. Correlation between VEN exposure and composite response (cCR)

Paragraph S1. Protocol details

a) Transfusion requirements: both platelet and red blood cell (RBC) transfusions were expressed in terms of transfusions/day, in order to normalize data for the duration of the cycle: the total number of transfusions performed during a cycle was divided for the duration of the cycle, expressed in days. For example, if the patient had performed 2 transfusions during a 28-day cycle, the transfusions/day parameter is 2/28=0.071.

b) Outpatients with severe neutropenia were admitted or strictly monitored in day hospital; however, if daily blood counts were not available, the approximation rules utilized to calculate the duration of neutropenia are as follows:

1) in the presence of a blood count with neutrophils (N) <500, without daily monitoring, the duration of neutropenia was calculated from the date of the first blood count with N< 500 to the last blood count with N<500, adding 1 day before and 1 day after.
2) in the presence of a blood count with N <100, without daily monitoring, the duration of neutropenia was calculated from the date of the first blood count with N< 100 to the last blood count with N<100, adding 2 days before and 2 days after.

These approximations were applied to avoid falsely short periods of aplasia in those rare neutropenic patients who were not strictly monitored in the outpatient setting. The approximations were decided based on the neutrophil count trends observed in frequently monitored neutropenic patients.

c) If the patient had 2 or more phases of neutropenia during a single cycle, the total duration was their sum.

d) antifungal agents were administered orally, according to their Summary of Product characteristics.

- posaconazole was administered in tablets, for prophylaxis
- voriconazole was administered when a fungal infection was deemed possible/probable
- isavuconazole was administered off-label in one patient, for prophylaxis, due to posaconazole toxicity.
- antifungal agents were sometimes started concurrently with venetoclax (VEN), sometimes before or after; however, we considered a VEN plasma determination to be under antifungal agents when the latter had been administered for at least 4 days.

Paragraph S2. Overall population, safety, and efficacy subsets

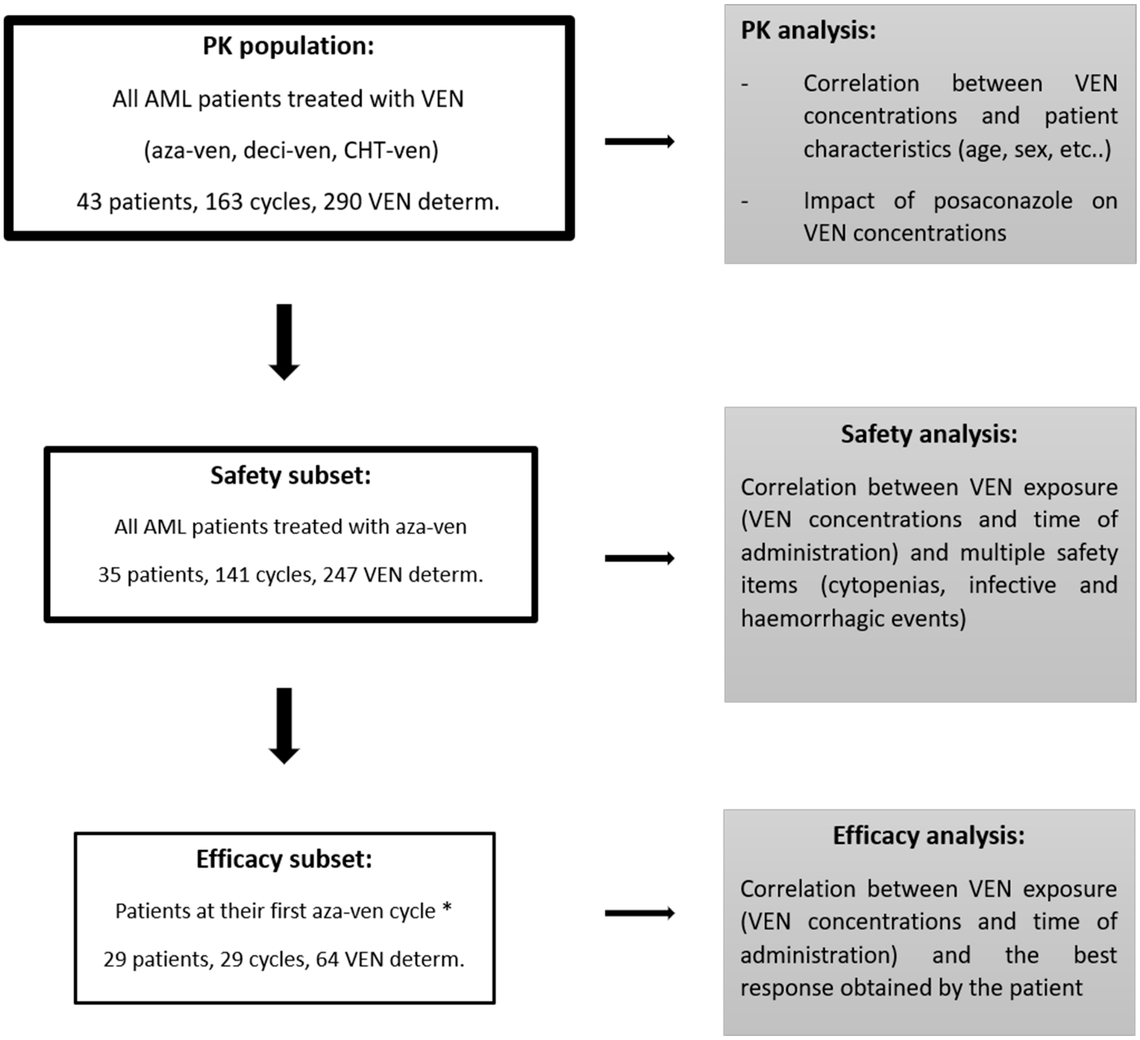

All VEN plasma concentrations were deemed valuable to perform a general PK analysis, correlating VEN concentrations to patients' characteristics (age, sex, etc.) and to explore the effect of posaconazole on VEN concentrations. For this reason, both patients treated with hypomethylating agents + VEN and patients treated with chemotherapy + VEN concur to create the PK population.

Conversely, the safety analysis was limited to patients treated with azacitidine and VEN (aza-ven), in order to avoid toxicity bias from chemotherapy and decitabine (safety subset).

The efficacy analysis considered only VEN determinations acquired during the first aza-ven cycle of untreated or R/R patients with >5% of blasts, for whom a bone marrow evaluation was available (efficacy subset). This choice was made because the median time to response to aza-ven is 1.3 months (according to the VIALE-A trial), meaning that the first cycle is the one with the greatest impact in inducing response. Below, a flow chart showing the subdivision in the different subsets.

Figure S1. Flow chart illustrating the whole study population and the different subsets. AML: acute myeloid leukemia; Aza-ven: azacitidine-venetoclax; deci-ven: decitabine-venetoclax; CHT: chemotherapy; VEN determ.: VEN plasma concentration determinations. Notes: *The efficacy subset only included patients with untreated or R/R AML with >5% of blasts, for whom a bone marrow evaluation was available. Patients that used aza-ven off-label for MRD eradication were excluded.

Paragraph S3. VEN plasma concentration analysis

S3.1 Chemicals

All reagents were of analytical grade. VEN (product code C5160) and VEN d7 (product code C8102), used as internal standard (IS), were purchased from Shimadzu Chemistry & Diagnostics (Illkirch Graffenstaden, Bas-Rhin, Grand Est, France). Acetonitrile (ACN) suitable for LC/MS (liquid chromatography/mass spectrometry), LiChrosolv®, product code 1.00029, formic acid 98%-100%, for analysis EMSURE ACS, Reag. Ph Eur, product code 1.00264, and dimethyl sulfoxide (DMSO) ACS reagent ≥99.9%, product code 472301, were purchased from Merck (Darmstadt, Germany). Ultrapure water (conductivity <0.05 µS/cm; ASTM parameter Type I) was obtained by using a 1720 Deionizer Device (G. Maina, Pecetto Torinese, Torino, Italy).

S3.2 Preparation of solvent and matrix-matched samples

Stock standard solutions of VEN and IS were prepared in DMSO, at 1 mg/mL. Working solutions were obtained by dilution in ACN (product code 1.00029, Merck) and stored at -20°C in the dark. Blank human plasma from healthy volunteers was spiked with the appropriate standard solutions to prepare matrix-matched samples (see below).

S3.3 Sample Preparation

The method was applied to the analysis of plasma samples collected in EDTA tubes. Following centrifugation, a 50 µL aliquot of plasma was transferred to a clean tube, and 1 mL of cold ACN (product code 1.00029, Merck) containing the IS at a concentration of 100 ng/mL was added for protein precipitation. The mixture was vortex-mixed and then stored at −20 °C for 10 min to enhance protein precipitation efficiency and obtain a cleaner supernatant prior to centrifugation [1,2]. Samples were subsequently centrifuged at 15,093 × *g*-force for 15 min at 23 °C, after which the clear supernatant was transferred to autosampler vials and injected into the LC-MS/MS system.

3.4 Instrumental analysis

The analysis of VEN plasma concentration was performed by liquid chromatography tandem mass spectrometry (LC-MS/MS). Chromatographic separation was achieved using a Nexera X2 liquid chromatograph (Sciex, Ontario, Canada), equipped with a vacuum degasser, a binary pump, an autosampler, and a column oven. Separation was carried out on a Phenomenex Luna Omega C18 column (1.6 µm, 100 Å, 100 × 2.1 mm), coupled with a Phenomenex UHPLC fully porous C18 guard column (2.1 mm) (Phenomenex, Bologna, Italy). The chromatographic run was performed using a binary mobile phase consisting of 0.1% (v/v) formic acid in water (A) and 0.1% (v/v) formic acid in ACN (B) under the following program: isocratic gradient with 60% of B in 0.5 min, linear gradient from 60% to 95% of B in 1.5 min, isocratic with 95% ACN for 0.5 minutes; total run time, included reconditioning time: 3.5 minutes. The injection volume was 1 µL, and the flow rate was 0.3 mL/min. Mass spectrometric detection was performed using a 6500 QTRAP mass spectrometer (Sciex) equipped with an electrospray ionization (ESI) source operating in positive ion mode. Data acquisition was carried out at unit mass resolution in selected reaction monitoring (SRM) mode. The optimized MS parameters were as follows:

curtain gas, 40 psi; collision gas, medium; ion spray voltage, 5500 V; source temperature, 400 °C; ion source gas 1, 50 psi; ion source gas 2, 50 psi; declustering potential, 200 V; entrance potential, 10 V. Two SRM transitions were monitored for VEN (m/z 868.2 → 321.0 and 868.2 → 636.2), and one for the IS (m/z 875.3 → 321.3). The collision energy (CE) and cell exit potential (CXP) values for the SRM transitions were set at 56, 38, and 56 eV (CE) and 21, 23, and 21 eV (CXP), respectively.

S3.5 Method Validation

All validation parameters generally required for quantitative bioanalytical procedures [3] were assessed, including selectivity, linearity, lower limit of quantification (LLOQ), accuracy, precision, and stability. In addition, validation parameters typically evaluated for LC–ESI–MS/MS methods were investigated, including the matrix effect (ME), defined as ion suppression or enhancement in ESI, and carry-over.

Six blank plasma samples were analyzed to assess selectivity. Potential interferences from endogenous substances were evaluated by inspecting the SRM chromatograms at the expected retention time of the analyte. No interfering signals ($S/N<3$) were observed at the analyte retention time in any of the blank samples, demonstrating that the method is selective for the analyte and free from positive interference from endogenous plasma components.

The calibration model was evaluated by analyzing three replicates of blank plasma spiked with the analyte at six concentration levels (0.25, 0.5, 1, 2, 5, and 10 mg/L) on three separate days. Calibration was performed using internal standardization. A blank sample was processed alongside the calibration series but was not included in the calibration curve. Calibration parameters were calculated using least-squares regression, applying a $1/x^2$ weighting factor. The LLOQ was defined as the lowest calibration level. Linearity was assessed over the stated range, yielding correlation coefficients (R^2) between 0.9952 and 0.9986.

Intra-run and inter-run accuracy (expressed as bias, %) and precision (expressed as coefficient of variation, CV%) were assessed at four concentration levels (0.25, 0.75, 4, and 7.5 mg/L), including the established LLOQ (0.25 mg/L), in accordance with EMA (European Medical Agency) guidelines. Each concentration level was analyzed in five replicates within a single analytical run and in three replicates across different days. Accuracy and precision were considered acceptable when bias% and CV% values did not exceed 15%, with an allowable limit of 20% at the LLOQ. All measured bias% and CV% values complied with these acceptance criteria.

ME was evaluated by comparing analyte responses in water and plasma matrices. ME was calculated as the percentage ratio between the chromatographic peak area of the analyte in plasma and that obtained in water. ME was also assessed for the IS at the concentration used for the analysis of real samples, and IS-normalized ME values were calculated. A slight matrix-induced signal suppression was observed (ME for VEN=83%; ME for IS=86%). The comparable extent of signal suppression between the analyte and the IS resulted in effective compensation, yielding accurate and reproducible quantitative determinations, as described above (IS-normalized ME=98%).

Carry-over was assessed by alternately injecting the highest matrix calibration standard and a blank sample; the absence of carry-over was confirmed when the signal-to-noise (S/N) ratio in the blank chromatogram was below 3 at the analyte's expected retention time.

The stability of standard solutions, matrix-matched samples and real plasma samples was also investigated, and all were found to be stable for at least six months.

Table S1. Effect of patient characteristics on VEN plasma concentration (Multivariate analysis)

Patient characteristics	Whole population (*p*-value)	No-AA group (*p*-value)	Pos group (*p*-value)
**GENDER**	0.347	0.646	0.487
**AGE**	0.429	0.274	0.927
**BMI**	0.415	0.113	0.473
**BSA**	0.086	0.108	0.197
**TREATMENT (deci-ven vs aza-ven)**	0.28	0.495	0.44
**TREATMENT (CHT-ven vs aza-ven)**	0.755	0.834	0.62
**Dose of VEN administration**	0.623	<0.001	0.003

Notes: BMI: Body Mass Index; BSA: Body Surface Area; aza-ven: azacitidine-venetoclax; deci-ven: decitabine-venetoclax; CHT-ven: chemotherapy-venetoclax. Analyses were obtained with a linear mixed effect model with a random intercept for the subject.

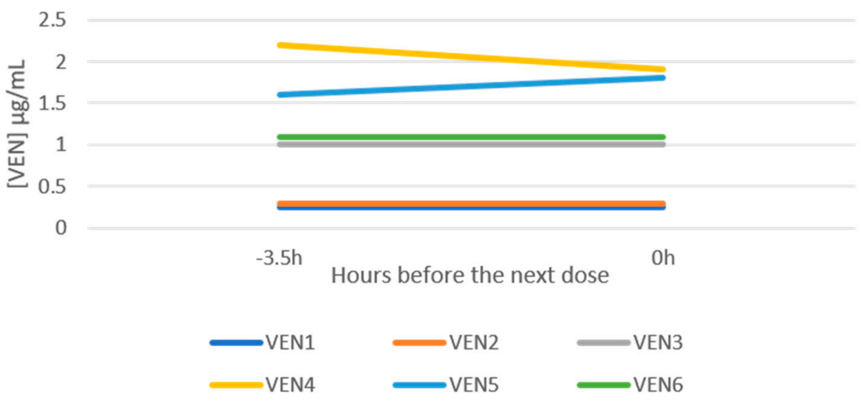

Figure S2. Comparison between approximated Cmin and true Cmin. Impact of a 3.5-hour delay in blood sampling at our Institution on 6 VEN plasma concentration determinations (VEN1, VEN2, VEN3, VEN4, VEN5, VEN6). VEN: venetoclax; [VEN]: venetoclax concentration; Cmin: trough concentration.

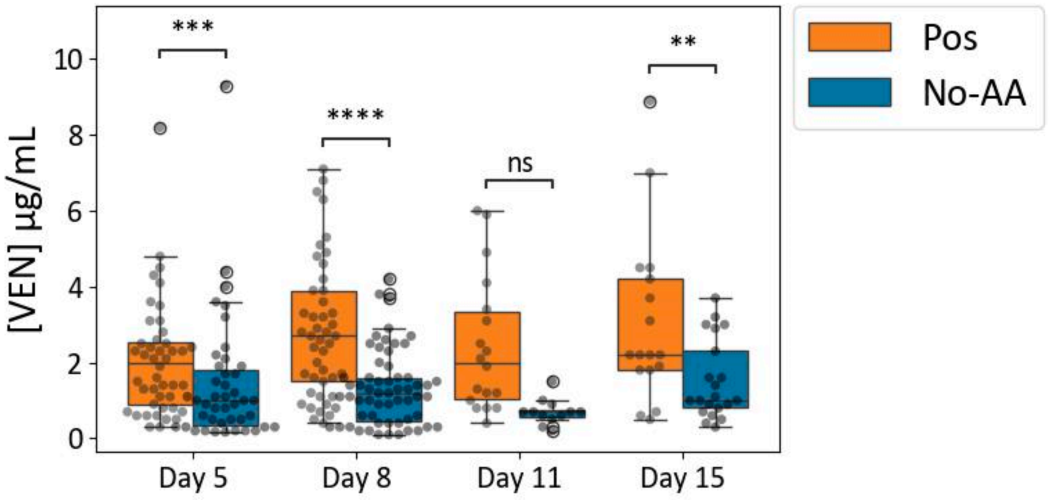

Figure S3. Comparison between Ven determinations at days 5, 8, 11 and 15 in the Pos group vs the No-AA group. [VEN]: venetoclax plasma concentration. ** p<0.01, ***p<0.001, ****p<0.0001, ns not significant. A linear mixed-effects model with random subject-specific intercepts and with the administered dose as covariate was used. Wald's tests were used to assess differences between timepoints.

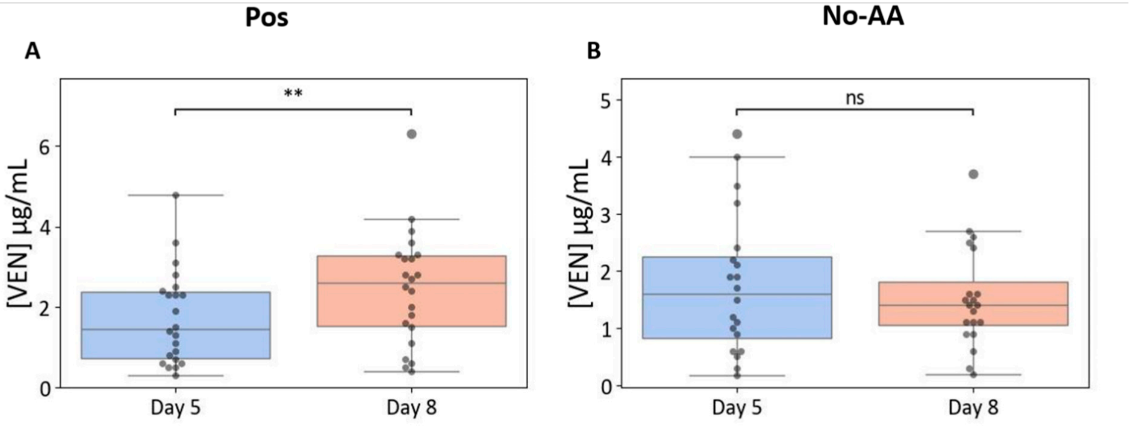

Figure S4. VEN accumulation between Day 5 and Day 8 in patients with both determinations available (paired data). Accumulation is significant in the Pos group **(A),** but not in the No-AA group **(B).** [VEN]: venetoclax plasma concentration. **p < 0.01, ns: not significant.

Venetoclax excretion in patients assuming posaconazole

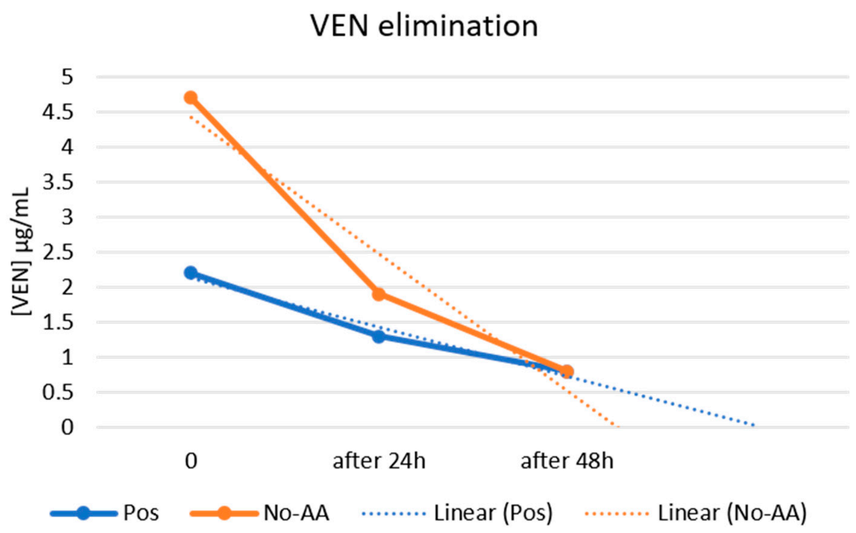

Figure S5. Comparison between VEN concentration decrease after withdrawal in the presence and in the absence of posaconazole. VEN concentrations were evaluated 24 and 48 hours after VEN missed dose (0); this analysis was performed in the same patient that underwent a cycle with posaconazole (50 mg/day of VEN) and another without (400 mg/day of VEN); in both cases, the steady state had already been achieved. The slopes of the lines (linear forecasts) show that VEN elimination is slower when posaconazole is co-administered.

Table S2. Calculated VEN T/2 in patients assuming posaconazole

**Patients**	**[VEN] (µg/mL) at withdrawal***	**[VEN] (µg/mL) +24h#**	**[VEN] (µg/mL) +48h#**	**[VEN] (µg/mL) +72h#**	**[VEN] (µg/mL) +240h#**	**Calculated T/2 (h)**
Pt1	4.1	3.3	1.8			40.4
Pt2	2.2	1.3	0.8			32.9
Pt3	1.9	0.8	0.5			24.9
Pt4	1.7	0.8	0.6			31.9
Pt5	6.3			3	0.4	59.9

Notes: Pt: patient. *Withdrawal: the day of the first missed dose or the day before. #: Hours following the first missed dose.

Table S3. VEN plasma concentrations after multiple days from withdrawal, in the Pos group

**Patients**	**[VEN] (µg/mL) at withdrawal***	**[VEN] (µg/mL) + 5 days#**	**[VEN] (µg/mL) + 7 days#**	**[VEN] (µg/mL) + 10 days#**
Pt5	6.3			0.4

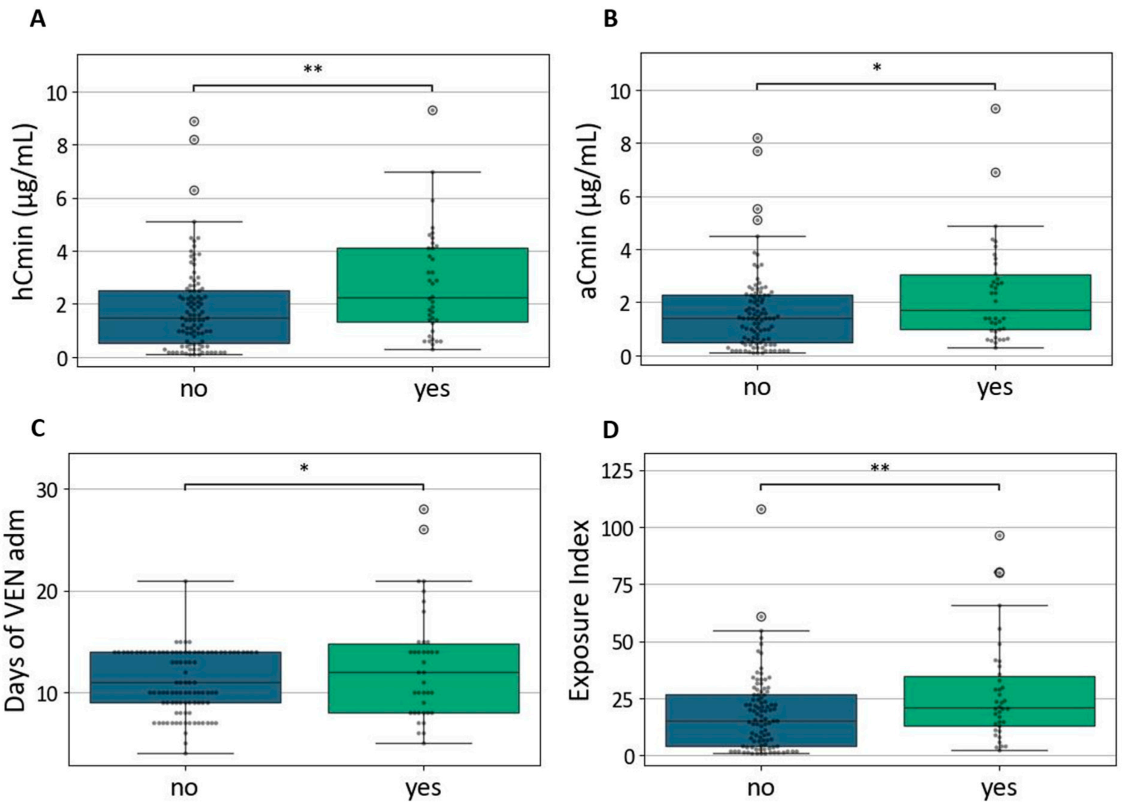

Pt6	4.7		0.2	
Pt7	4.5	0.2		
Pt8	8.9		1	

Notes: Pt: patient.*Withdrawal: the day of the first missed dose or the day before. #: days following the first missed dose.

Figure S6. Correlation between VEN exposure and profound neutropenia (N< 100/µL). N: neutrophils; hCmin: highest trough VEN concentration during each cycle; aCmin: average trough VEN concentration during each cycle; Days of VEN adm: days of VEN administration; Exposure index: product between aCmin and days of VEN administration. * $p< 0.05$, ** $p < 0.01$.

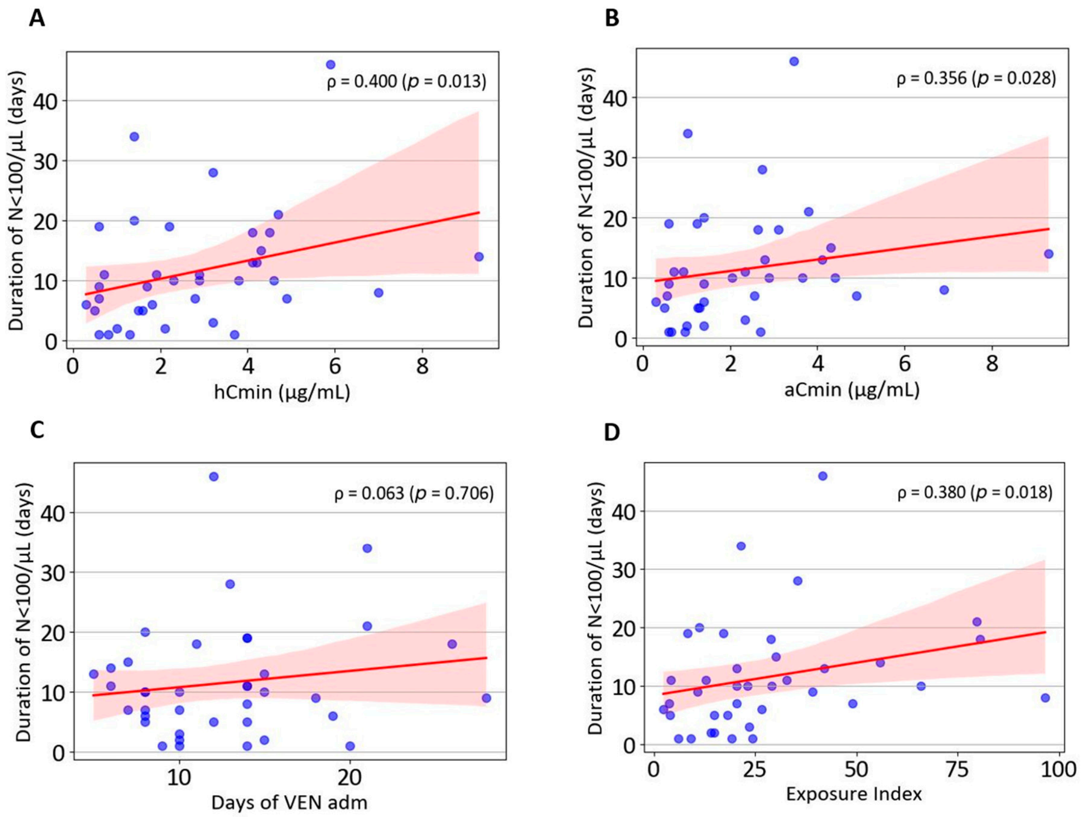

Figure S7. Correlation between VEN exposure and duration of profound neutropenia (days with N< 100/μL). N: neutrophils; hCmin: highest trough VEN concentration during each cycle; aCmin: average trough VEN concentration during each cycle; Days of VEN adm: days of VEN administration; Exposure index: product between aCmin and days of VEN administration. The duration of profound neutropenia was calculated only in patients undergoing N<100/mmc (i.e., duration of profound neutropenia>0). The value of Spearman's ρ coefficient and the corresponding p-value are displayed.

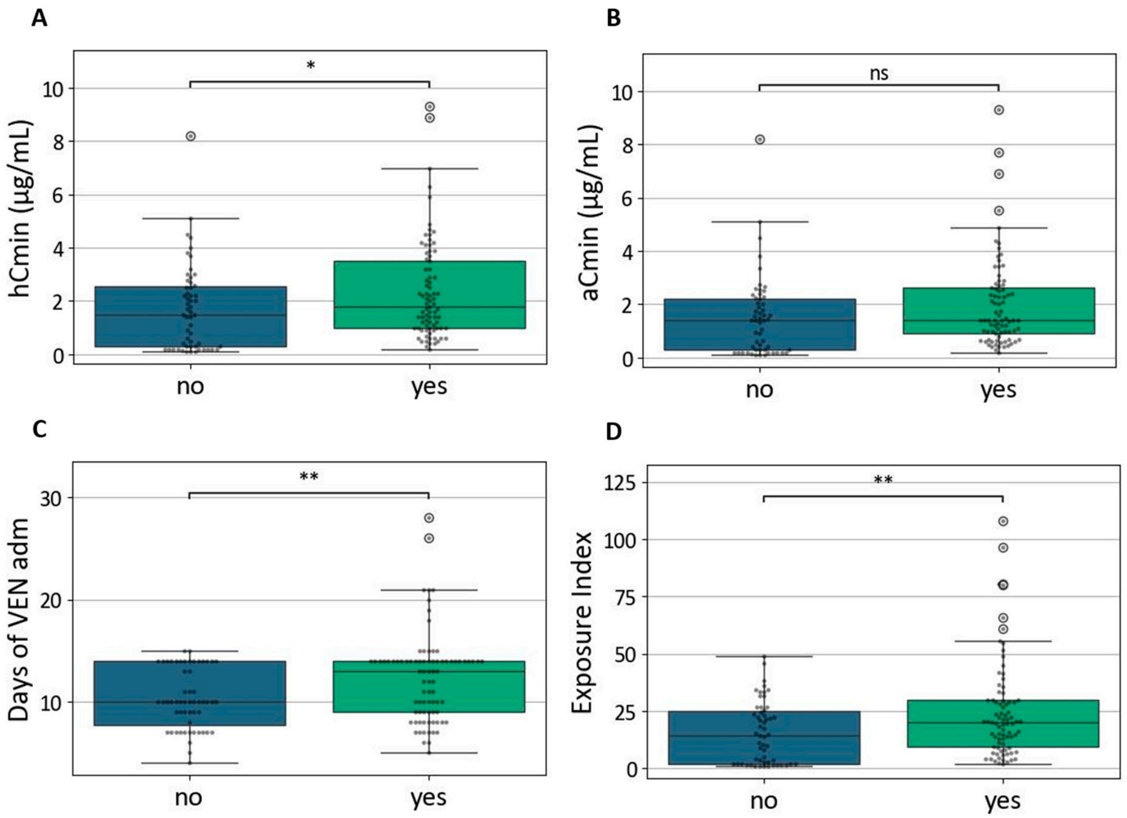

Figure S8. Correlation between VEN exposure and severe neutropenia (N<500/µL). N: neutrophils; hCmin: highest trough VEN concentration during each cycle; aCmin: average trough VEN concentration during each cycle; Days of VEN adm: days of VEN administration; Exposure index: product between aCmin and days of VEN administration. * $p < 0.05$, ** $p < 0.01$, ns not significant.

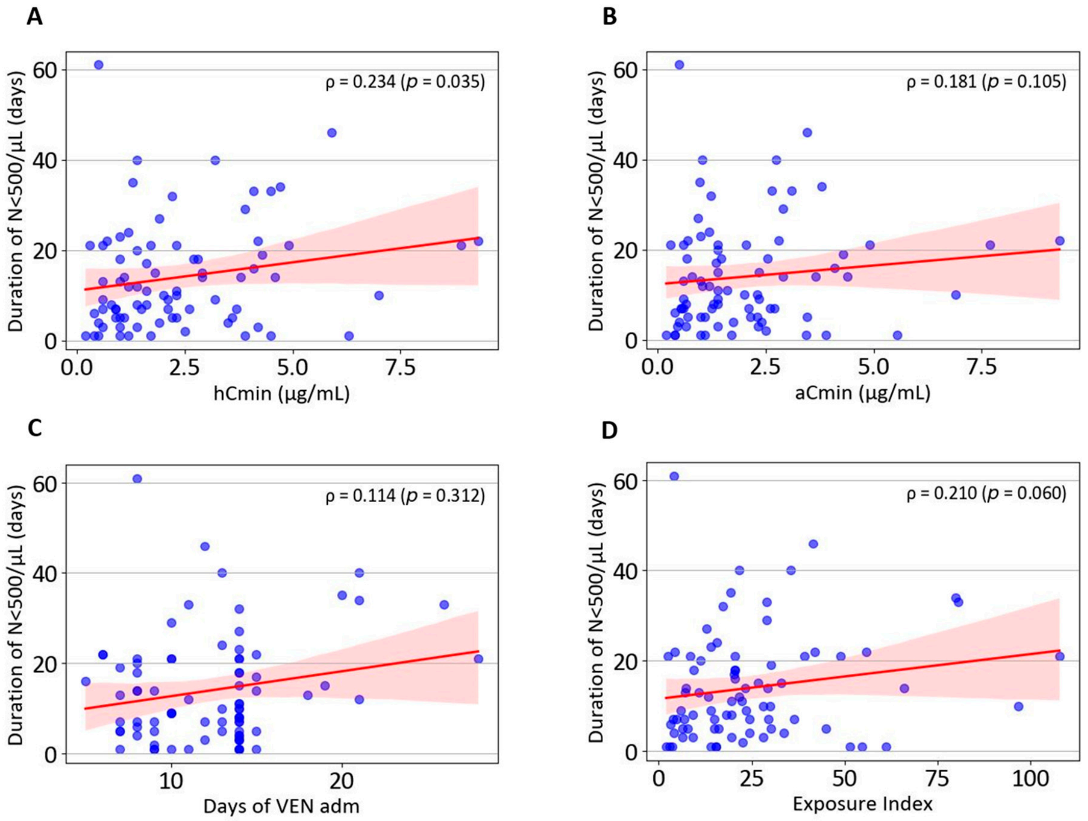

Figure S9. Correlation between VEN exposure and duration of severe neutropenia (days with N<500/µL). N: neutrophils; hCmin: highest trough VEN concentration during each cycle; aCmin: average trough VEN concentration during each cycle; Days of VEN adm: days of VEN administration; Exposure index: product between aCmin and days of VEN administration. The duration of severe neutropenia was calculated only in patients undergoing N< 500/mmc (i.e., duration of severe neutropenia>0). The value of Spearman's ρ coefficient and the corresponding *p*-value are displayed.

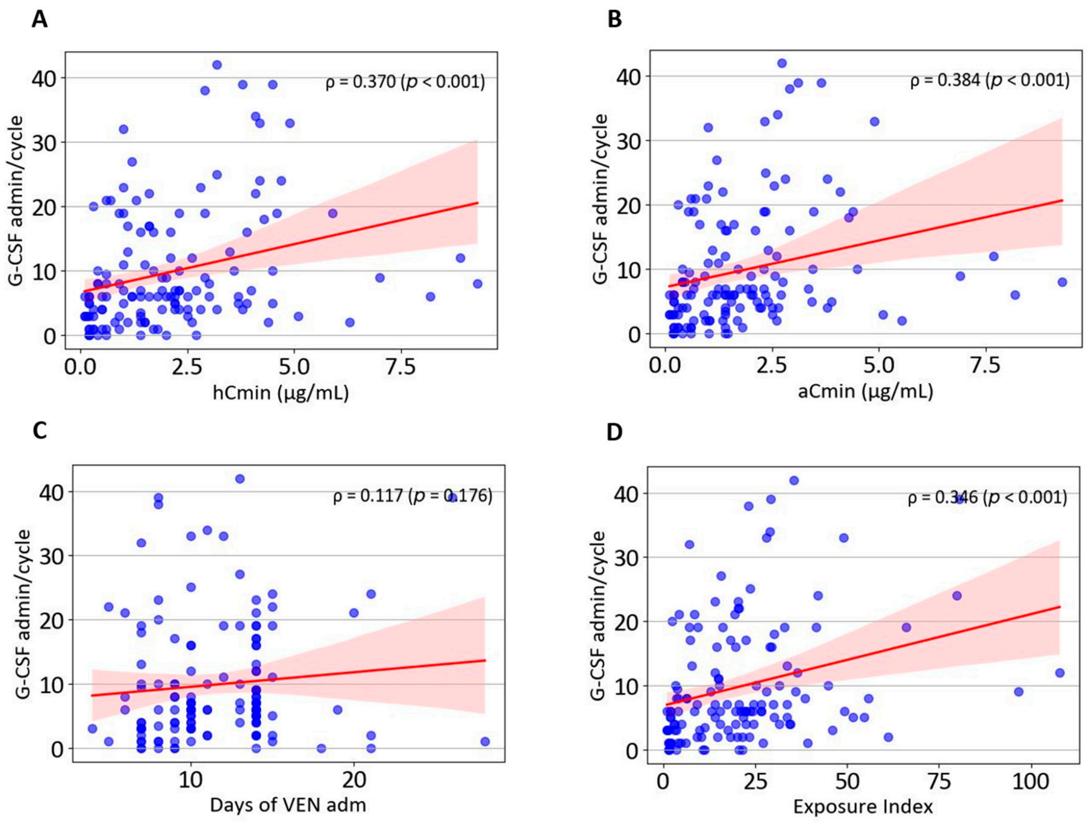

Figure S10. Correlation between VEN exposure and G-CSF requirement during each cycle. G-CSF: granulocyte colony-stimulating factor. hCmin: highest trough VEN concentration during each cycle; aCmin: average trough VEN concentration during each cycle; Days of VEN adm: days of VEN administration; Exposure index: product between aCmin and days of VEN administration. The value of Spearman's ρ coefficient and the corresponding *p*-value are displayed.

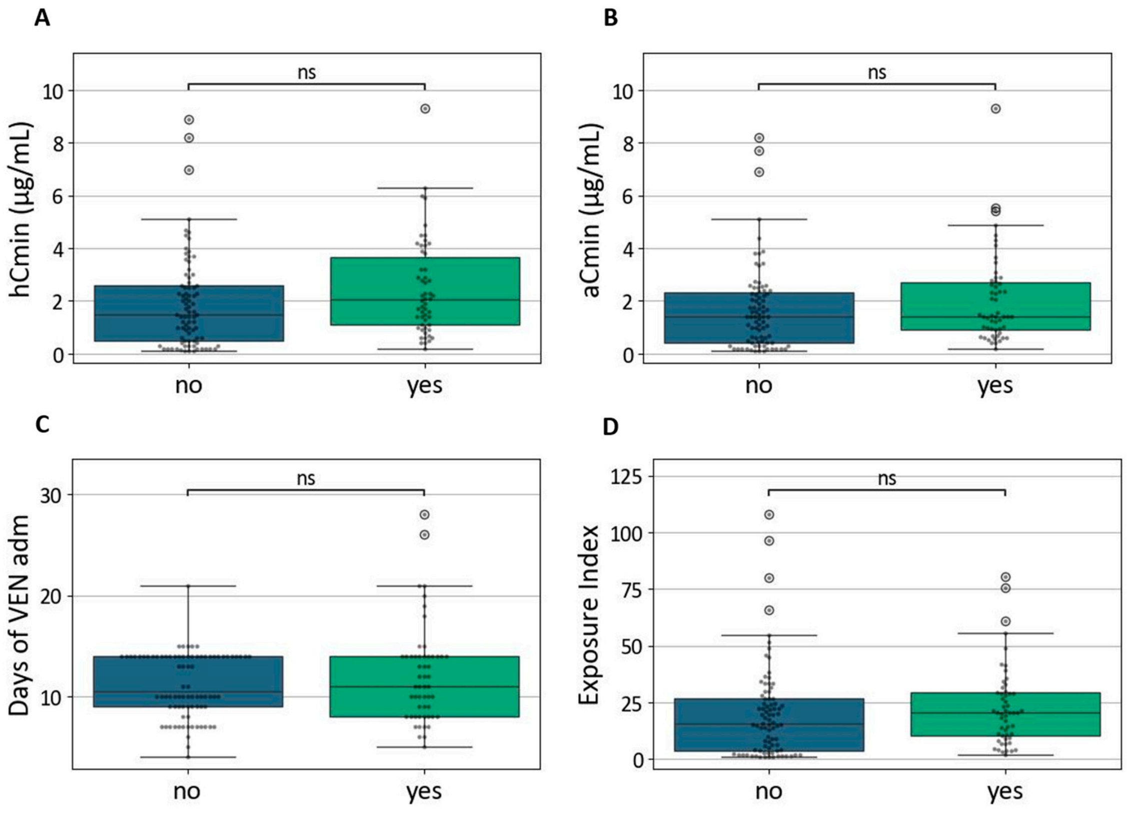

Figure S11. Correlation between VEN exposure and grade 4 thrombocytopenia (platelets <25.000/µL). hCmin: highest trough VEN concentration during each cycle; aCmin: average trough VEN concentration during each cycle; Days of VEN adm: days of VEN administration; Exposure index: product between aCmin and days of VEN administration. ns: not significant.

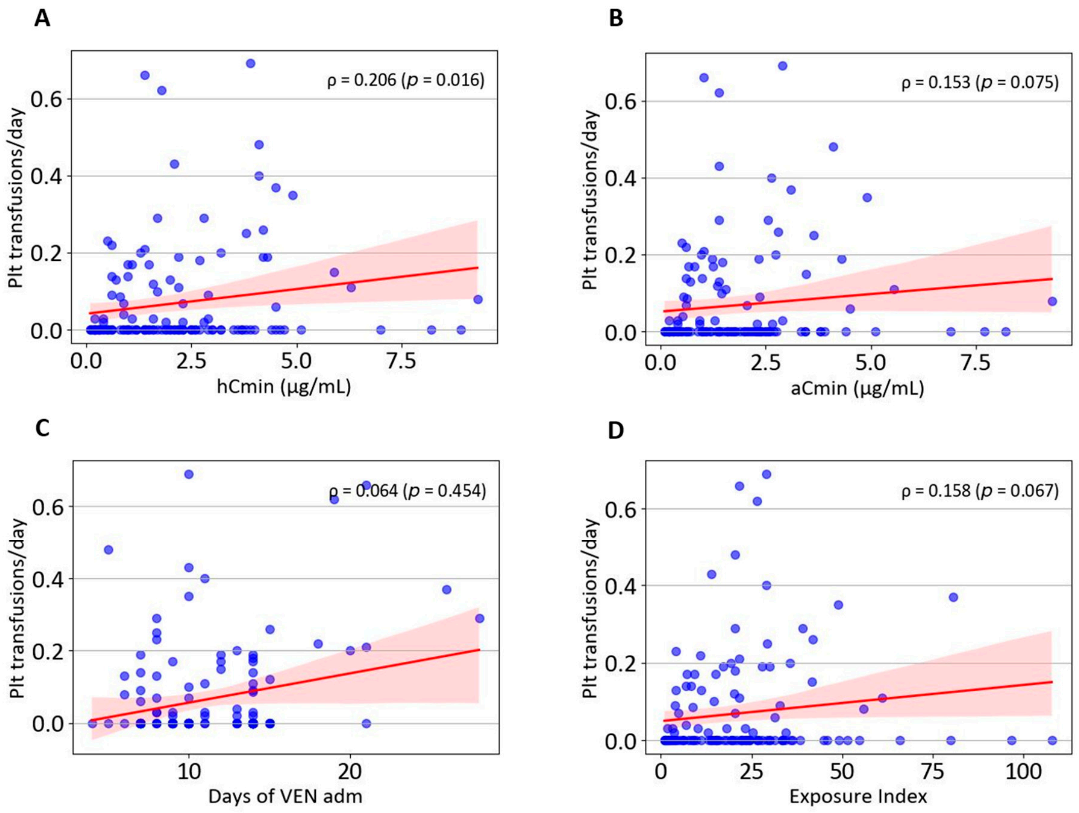

Figure S12. Correlation between VEN exposure and platelet transfusion requirements. Plt: platelet; hCmin: highest trough VEN concentration during each cycle; aCmin: average trough VEN concentration during each cycle; Days of VEN adm: days of VEN administration; Exposure index: product between aCmin and days of VEN administration. Platelet transfusions were expressed in terms of transfusions/day, in order to normalize data for the duration of the cycle (please refer to paragraph S1). The value of Spearman's ρ coefficient and the corresponding *p*-value are displayed.

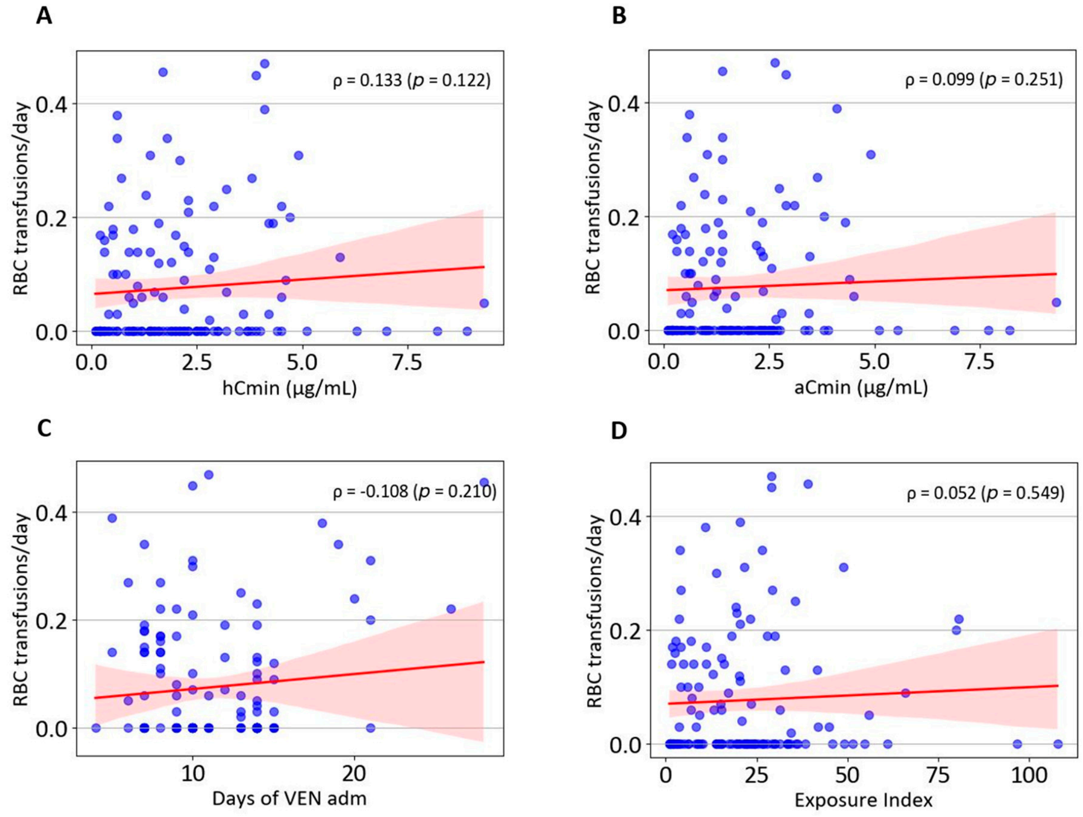

Figure S13. Correlation between VEN exposure and RBC transfusion requirements. RBC: red blood cells; hCmin: highest trough VEN concentration during each cycle; aCmin: average trough VEN concentration during each cycle; Days of VEN adm: days of VEN administration; Exposure index: product between aCmin and days of VEN administration. RBC transfusions were expressed in terms of transfusions/day, in order to normalize data for the duration of the cycle (please refer to paragraph S1). The value of Spearman's ρ coefficient and the corresponding *p*-value are displayed.

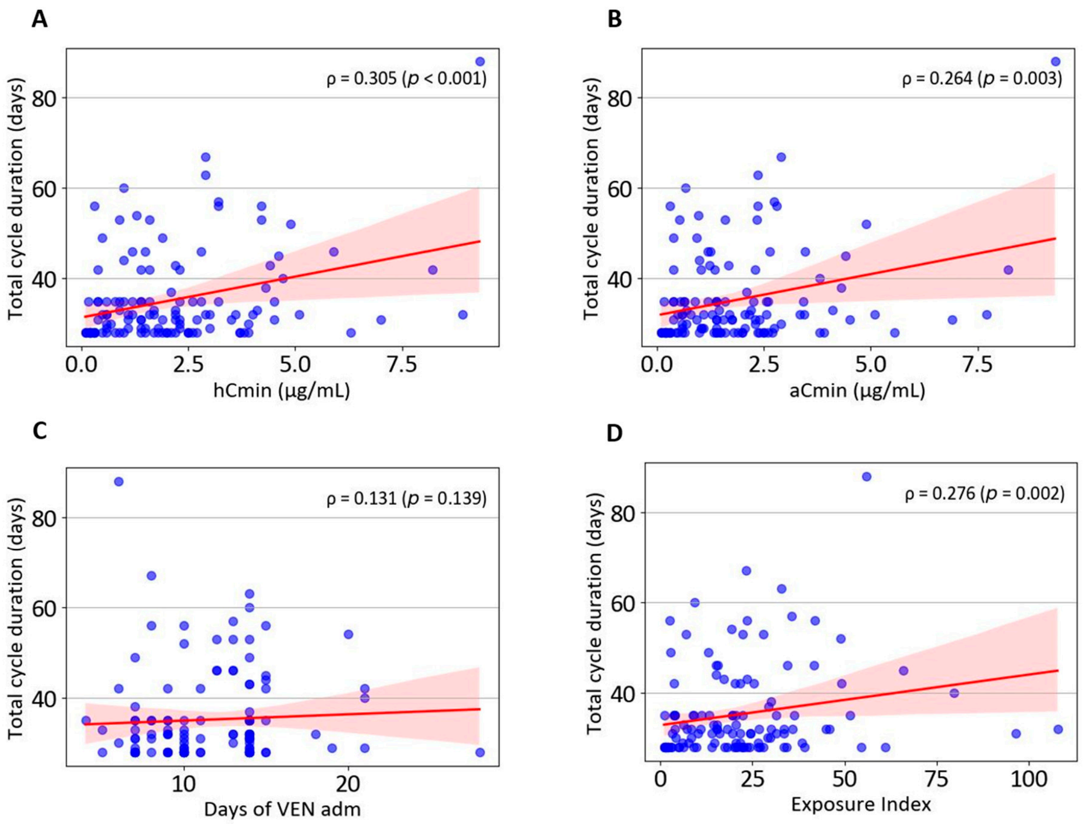

Figure S14. Correlation between VEN exposure and total cycle duration. hCmin: highest trough VEN concentration during each cycle; aCmin: average trough VEN concentration during each cycle; Days of VEN adm: days of VEN administration; Exposure index: product between aCmin and days of VEN administration. The value of Spearman's ρ coefficient and the corresponding *p*-value are displayed.

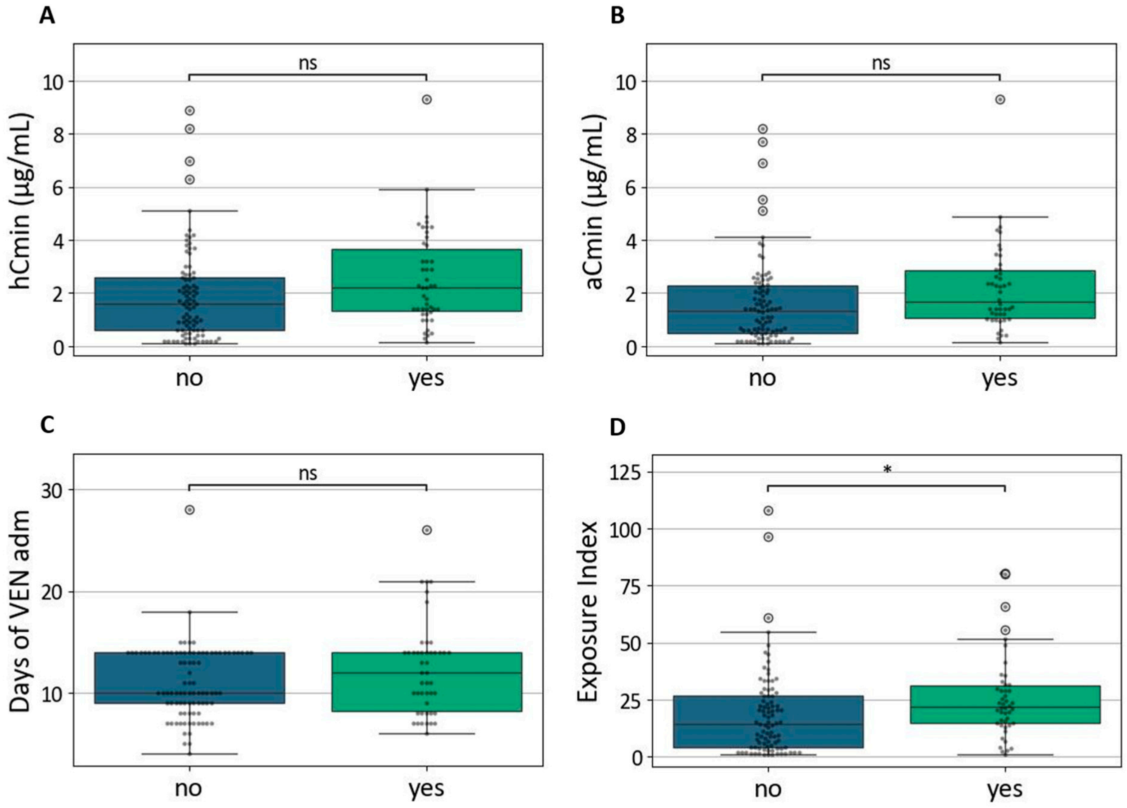

Figure S15. Correlation between VEN exposure and infective adverse events. hCmin: highest trough VEN concentration during each cycle; aCmin: average trough VEN concentration during each cycle; Days of VEN adm: days of VEN administration; Exposure index: product between aCmin and days of VEN administration. * $p < 0.05$, ns: not significant.

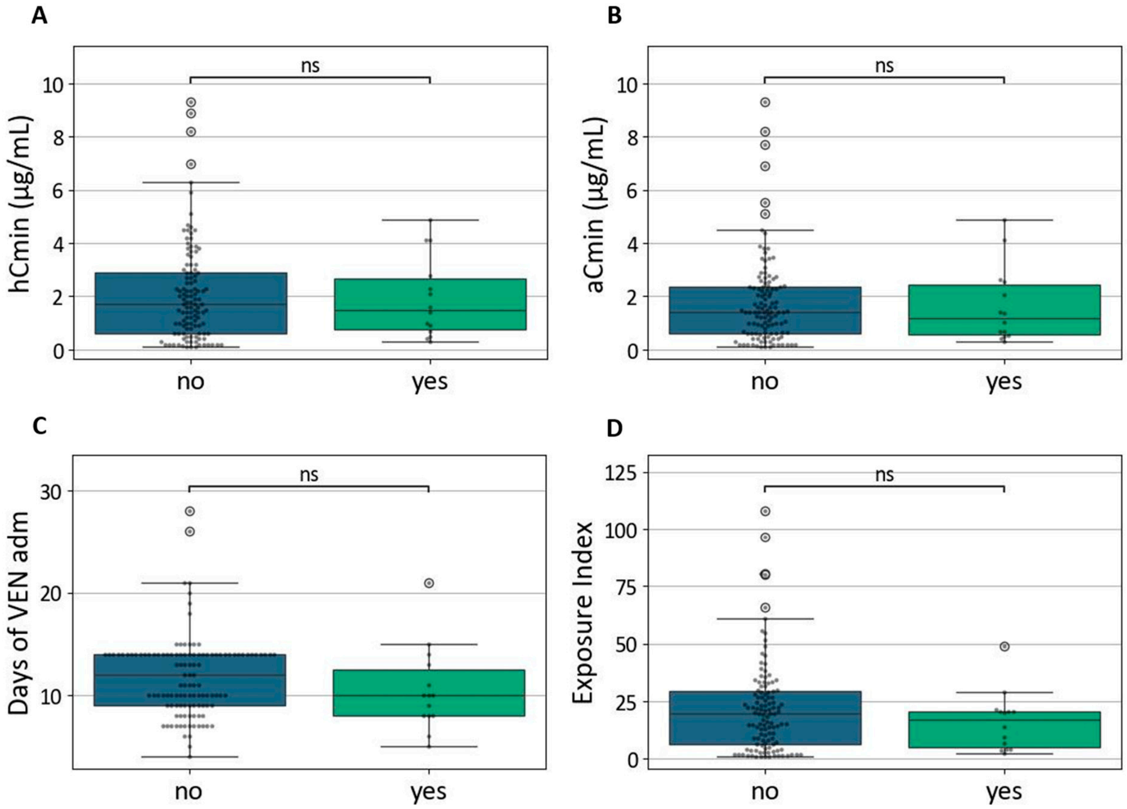

Figure S16. Correlation between VEN exposure and bleeding adverse events. hCmin: highest trough VEN concentration during each cycle; aCmin: average trough VEN concentration during each cycle; Days of VEN adm: days of VEN administration; Exposure index: product between aCmin and days of VEN administration; ns: not significant.

Safety outcomes according to the 0.5-4 μg/mL range

Table S4. Proportion of cycles with VEN concentrations always within the 0.5-4 μg/mL range vs cycles with out-of-range values

Cycles with:	**Percentage %**
- all VEN determinations in the 0.5-4 μg/mL range	66.3%
- at least one VEN determination < 0.5μg/mL	14.1%
- at least one VEN determination > 4 μg/mL	19.6%

Table S5. Comparison of safety outcomes in cycles with VEN concentrations within the 0.5-4 μg/mL range *vs* cycles with at least one value above or below

Safety parameters	< 0.5μg/mL *vs.* 0.5-4 μg/mL (Coeff, *p*-value)	> 4 μg/mL *vs.* 0.5-4 μg/mL (Coeff, *p*-value)
**N < 100/μL**	-1.71 (0.026)	1.24 (0.017)
**Duration of N<100/μL (including duration of neutropenia =0 days)**	-1.76 (0.215)	7.04 (< 0.001)
**Duration of N<100/μL (only when duration of neutropenia>0 days)**	3.38 (0.616)	7.52 (0.029)
**N < 500/ μL**	-1.06 (p 0.016)	0.81 (0.18)
**Duration of N<500/μL (including duration of neutropenia =0 days)**	-5.05 (0.03)	7.19 (0.01)
**Duration of N<500/μL (only when duration of neutropenia>0 days)**	-4.33 (0.262)	6.31 (0.065)
**G-CSF admin/cycle**	-4.45 (0.019)	7.1 (0.002)
**Plts<25.000/μL**	-0.77 (0.109)	0.8 (0.105)
**Plt transfusions/day**	-0.05 (0.062)	0.07 (0.038)
**RBC transfusions/day**	-0.036 (0.135)	0.04 (0.112)
**Total cycle duration**	-0.69 (0.739)	8.43 (0.001)
**Infective AEs**	-1 (0.063)	0.71 (0.16)
**Bleeding AEs**	0.53 (0.43)	0.79 (0.29)

Notes: The duration of neutropenia was calculated both in the whole safety subset and only in patients actually undergoing N< 100 or 500/μL (i.e., duration of neutropenia>0 days). Highlighted values are statistically significant ($p<0.05$). The regression coefficient and corresponding p-value were obtained from a logistic regression (for binary events) or a linear regression (for continuous events), using the event as response variable and concentration-range (below, inside or above the range) as explanatory variable. Notes: Coeff: regression coefficient; N: neutrophils; G-CSF: granulocyte colony-stimulating factor; Plt: platelets; RBC: red blood cells; AE: adverse event.

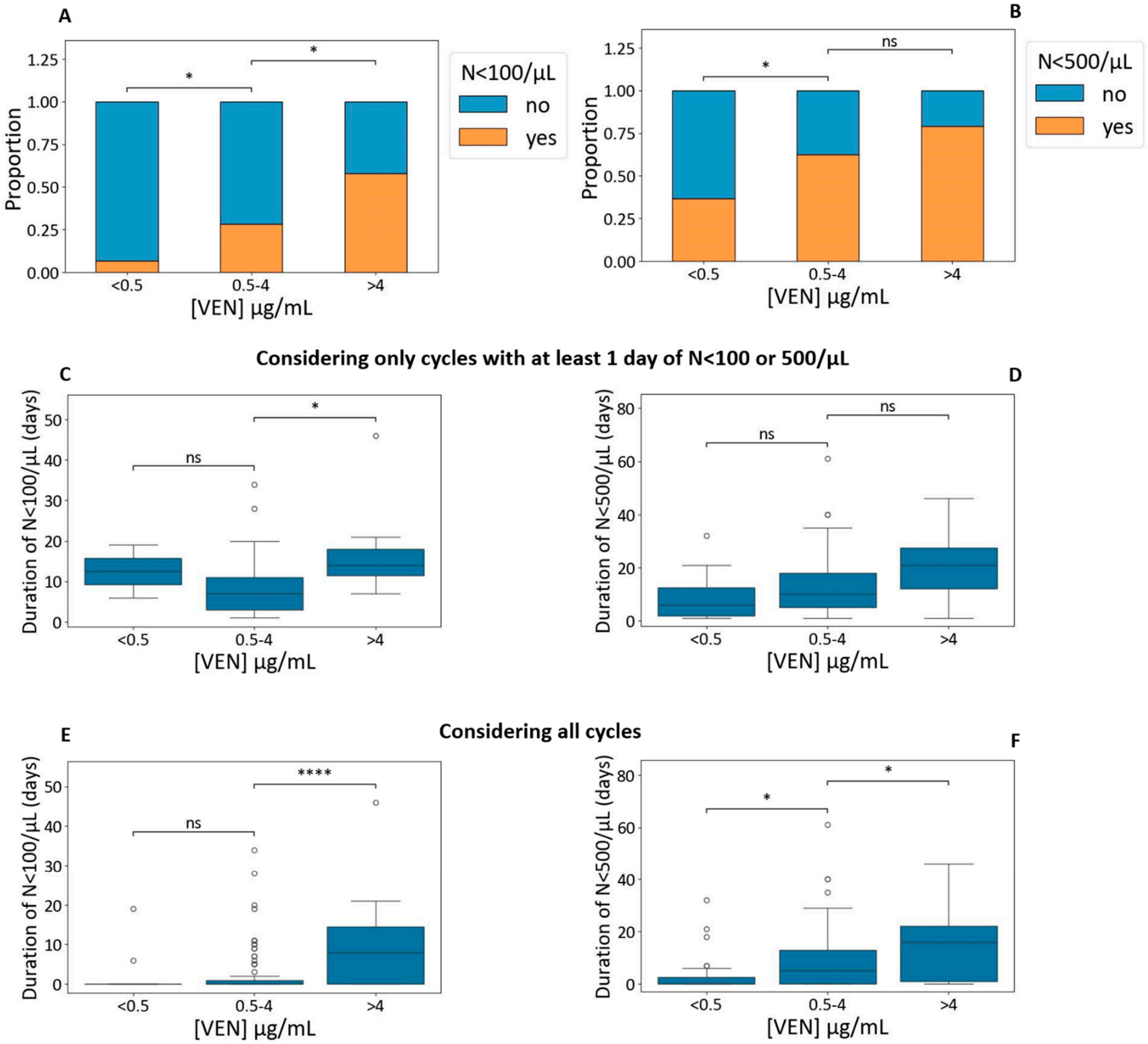

A
B
Proportion
N<100/μL
N<500/μL
no
yes
[VEN] μg/mL
<0.5
0.5-4
>4
*
ns
Considering only cycles with at least 1 day of N<100 or 500/μL
C
D
Duration of N<100/μL (days)
Duration of N<500/μL (days)
Considering all cycles
E
F
****

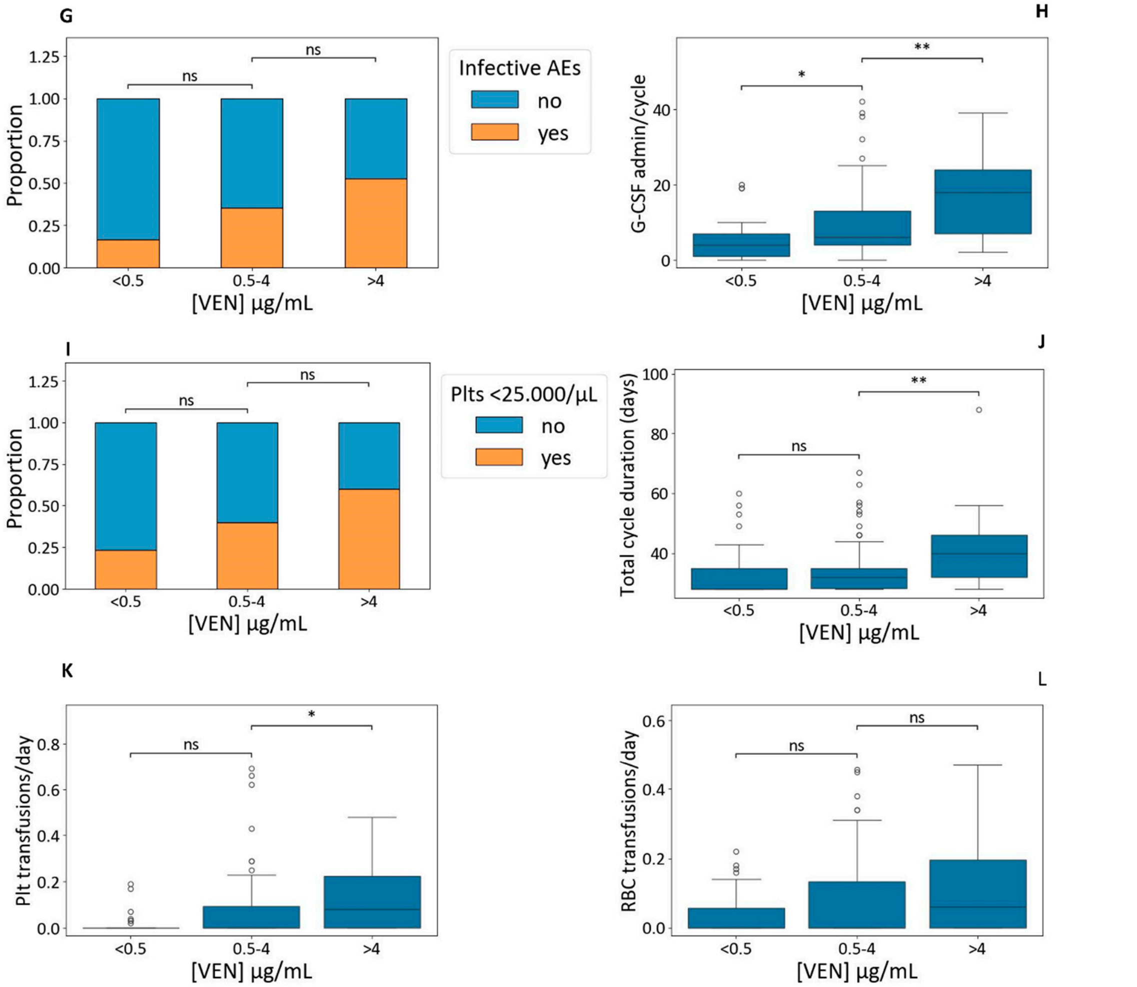

Figure S17. Comparison of multiple safety outcomes in cycles with VEN concentrations within the 0.5-4 µg/ml range *vs* cycles with at least one value above or below. (A) Profound neutropenia (N< 100/µL); (B) Severe neutropenia (N<500/µL); (C) Duration of N< 100/µL considering only cycles with at least 1 day with N< 100/µL; (D) Duration of N< 500/µL considering only cycles with at least 1 day with N< 500/µL; (E) Duration of N< 100/µL considering all cycles; (F) Duration of N< 500/µL considering all cycles; (G) Infective adverse events; (H) G-CSF need; (I) Plts<25.000/µL; (J) Total cycle duration; (K) Plt transfusions; (L) RBC transfusions. [VEN]: venetoclax plasma concentration; N: neutrophils; G-CSF: granulocyte colony-stimulating factor; Plt: platelets; RBC: red blood cells. AE: adverse events. *p<0.05, **p<0.01, ****p<0.0001, ns: not significant.

Table S6. Response rates in the whole efficacy subset and in specific subgroups.

AML Category	Patients, n (%)	ORR, n (%)	cCR, n (%)	NonR, n (%)
**Total (Efficacy subset)**	29 (100%)	20/29 (69%)	16/29 (55%)	9/29 (31%)
**Untreated *vs.* R/R**				
Untreated	23/29 (79%)	16/23 (70%)	13/23 (57%)	7/23 (30%)
R/R	6/29 (21%)	4/6 (67%)	3/6 (50%)	2/6 (33%)
***De novo vs.* secondary**				
De novo	13/29 (45%)	10/13 (77%)	10/13 (77%)	3/13 (23%)
Secondary	16/29 (55%)	10/16 (63%)	6/16 (38%)	6/16 (38%)
**Therapy-related**				
Therapy-related	3/29 (10%)	0/3 (0%)	0/3 (0%)	3/3 (100%)
Non therapy-related	26 /29 (90%)	20/26 (77%)	16/26 (62%)	6/26 (23%)
**ELN 2022 risk**				
Favorable	4/29 (13.8%)	4/4 (100%)	4/4 (100%)	0/4 (0%)
Intermediate	9/29 (31%)	7/9 (78%)	7/9 (78%)	2/9 (22%)
Adverse	16/29 (55.2%)	9/16 (56%)	5/16 (31%)	7/16 (44%)
**ELN 2024 risk**				
Favorable	4/29 (14%)	4/4 (100%)	3/4 (75%)	0/4 (0%)
Intermediate	18/29 (62%)	13/18 (72%)	11/18 (61%)	5/18 (28%)
Adverse	7/29 (24%)	3/7 (43%)	2/7 (29%)	4/7 (57%)

Notes: ORR: overall response rate, including CR (complete response), CRi (CR with incomplete hematologic recovery) and PR (partial response). cCR: composite response, including CR and CRi. NonR: non responders.

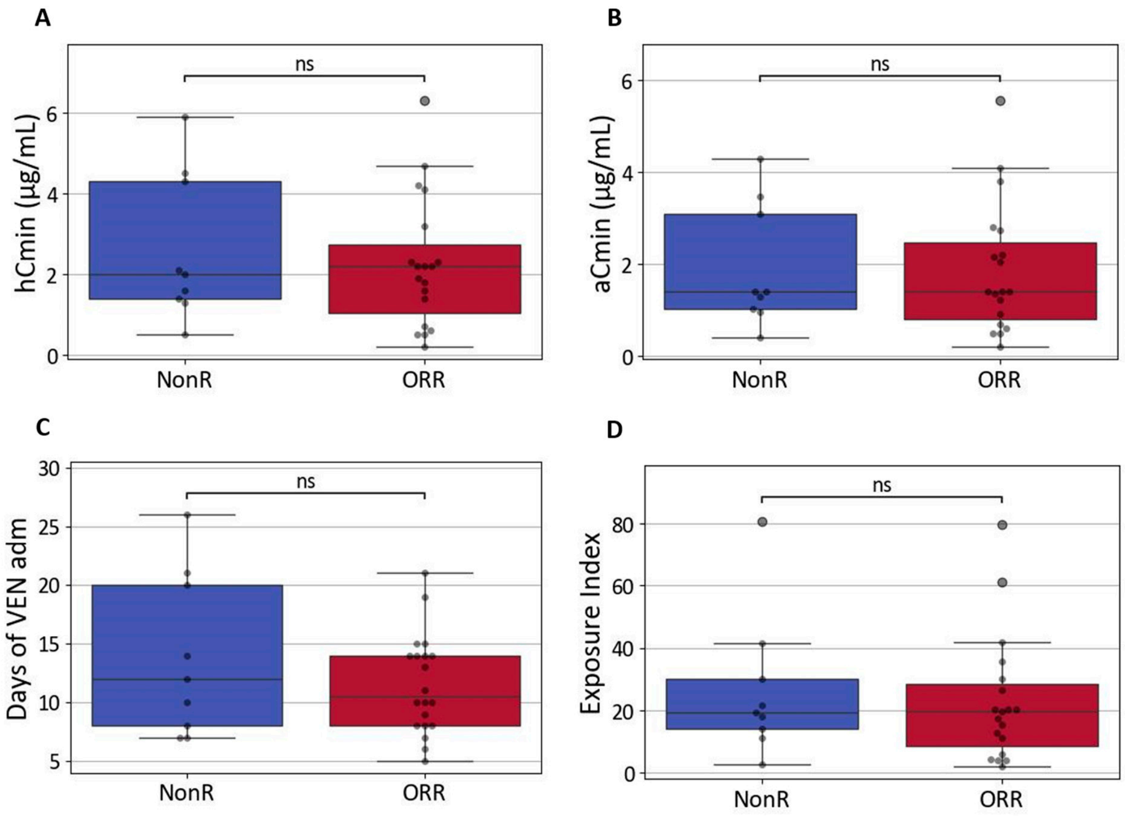

Figure S18. Correlation between VEN exposure and overall response rate (ORR). hCmin: highest trough VEN concentration during each cycle; aCmin: average trough VEN concentration during each cycle; Days of VEN adm: days of VEN administration; Exposure index: product between aCmin and days of VEN administration. NonR: non response. ORR: overall response rate, including PR (partial response), CR (complete remission) and CRi (CR with incomplete hematologic recovery); ns: not significant.

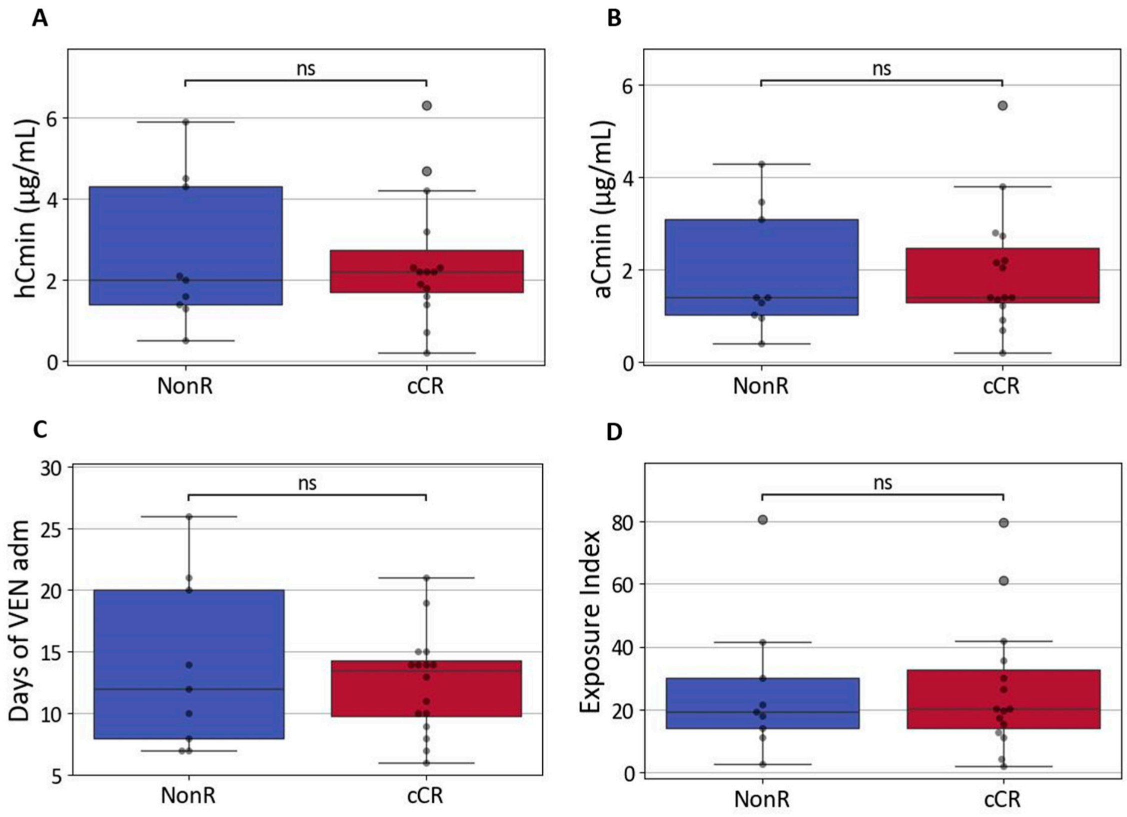

Figure S19. Correlation between VEN exposure and composite response (cCR). hCmin: highest trough VEN concentration during each cycle; aCmin: average trough VEN concentration during each cycle; Days of VEN adm: days of VEN administration; Exposure index: product between aCmin and days of VEN administration. NonR: non response. cCR: composite response, including CR (complete remission) and CRi (CR with incomplete hematologic recovery). ns: not significant.

Supplementary References

[1] Polson, Cara, et al. "Optimization of protein precipitation based upon effectiveness of protein removal and ionization effect in liquid chromatography–tandem mass spectrometry." *Journal of Chromatography B* 785.2 (2003): 263-275.

[2] Qin, Chaolong, et al. "Development and validation of a cost-effective and sensitive bioanalytical HPLC-UV method for determination of lopinavir in rat and human plasma." *Biomedical Chromatography* 34.11 (2020): e4934.

[3] EMA/CHMP/ICH/172948/2019; ICH Guideline M10 on Bioanalytical Method Validation. European Medicines Agency: Amsterdam, The Netherlands, 2019
